# Toughening PLA via Diisocyanate-Induced Dynamic Vulcanization of Carboxylated Oleic Acid: Role of Crosslinked Network Topology

**DOI:** 10.3390/polym18182297

**Published:** 2026-09-19

**Authors:** Dongmei Xie, Xiao Li, Jiaqi Cai, Xiaodi Mao, Hongzhi Liu, Ping Zhang

**Affiliations:** School of Materials Science and Engineering, NingboTech University, Ningbo 315100, China

**Keywords:** polylactic acid, dynamic vulcanization, toughening, cross-linked, topology

## Abstract

Diisocyanate-induced dynamic vulcanization of difunctional fatty acids has emerged as a universal strategy to efficiently improve the impact resistance of polylactic acid (PLA). However, how the crosslinked network topology of the in situ formed polyamide elastomer (COPA) affects its toughening efficacy on PLA remains unknown. Here, we synthesized two carboxylated fatty acids with distinct molecular architectures (TCOA and NCOA) from technical-grade and high-purity oleic acids via UV-initiated thiol-ene click chemistry. These two diacids, along with tetradecanedioic acid (TA) without a dangling chain, were dynamically vulcanized with hexamethylene diisocyanate (HDI) to toughen PLA. By varying NCO/COOH molar ratios between HDI and TCOA, their effects on gel content, crosslinking density, phase morphology, and mechanical properties of resulting blends were systematically investigated. With increasing the ratio, both gel content and interfacial adhesion with PLA in the blends were enhanced, accompanied by the transformation of phase structure from “sea-island” morphology to a partially or fully co-continuous one. At a molar ratio of 1.8:1, the notched impact strength value of the blend reached 86.5 kJ/m^2^. By substituting TCOA with high-purity NCOA or TA, comparable gel content and interfacial compatibilization level, and co-continuous morphologies were achieved. Notably, NCOA yielded a PLA blend with a remarkably higher impact toughness (131.0 kJ/m^2^). The linear chain structure of TA led to a higher crosslinking density of the formed TAPA domains, which unfavorably suppressed their cavitation during impact fracture and thus resulted in an inferior impact strength (7.0 kJ/m^2^). These findings provide valuable insights into the toughening mechanism of PLA via dynamic vulcanization of monomers.

## 1. Introduction

Considering depleting petroleum reserves and mounting environmental concerns, bio-based polymers have garnered significant interest [1,2,3]. Polylactic acid (PLA), an aliphatic polyester derived from renewable feedstocks, is renowned for its excellent biodegradability, biocompatibility, and mechanical properties [4,5,6,7]. Consequently, it is highly sought after for numerous applications across automotive, electronics, biomedical, and food packaging sectors [8,9,10,11]. The application of PLA in fields requiring high impact resistance is severely restricted by its inherent brittleness, as evidenced by its low notched impact strength (IS) [12,13,14].

Due to the unfavorable compatibility [15], direct melt-blending of PLA with elastomers or rubbers usually fails to obtain the favorable impact toughness. Several strategies have been developed to improve interfacial compatibility between PLA and toughening agents and thus to promote impact toughness of resulting blends.

Among various strategies, dynamic vulcanization has become one of the most promising ones for fabricating super-tough PLA materials in recent decades [16]. Different from rubber-toughened PLA blends that only involve interfacial compatibilization between the toughening phase and PLA, dynamic vulcanization not only yields the crosslinking of the rubbery phase but also enables the occurrence of in situ interfacial compatibilization, thereby eliminating the need for an additional compatibilizer. At present, the toughening agents have extended from petroleum-based elastomers or rubbers to bio-based counterparts, such as thermoplastic polyurethane [5,17], natural rubber (NR) [18,19,20,21], epoxidized natural rubber (ENR) [22,23,24,25], nitrile butadiene rubber (NBR) [26,27], polyesters [28,29,30,31], epoxidized soybean oil precursors cured with sebacic acid (VESO) [32], and polyisocyanate/polyol systems [33,34,35], etc.

Compared to cross-linkable elastomers or rubbers, reactive monomers, including small molecules and low-molecular-weight precursors, have recently emerged as more promising toughening agents owing to low viscosity, flexible design and high reactivity. During dynamic vulcanization, reactive monomers can simultaneously undergo polymerization, crosslinking, and reactive compatibilization. Therefore, the morphology of the toughening phase can be readily tuned by altering molar ratios of functional groups between complementary monomers. To preserve the bio-based nature of PLA materials, some bio-based monomers have been employed for dynamic vulcanization, such as epoxidized soybean oil (ESO) [36,37], castor oil (CO) [38,39], and plant oil-derived dimer acid [40,41]. In a recent work by our group, a fully bio-based super-tough PLA blend was fabricated by employing excess difunctional L-lysine diisocyanate (LDI) to trigger dynamic vulcanization of hydrogenated dimer acid (HDA) [40]. The maximum notched IS up to 109.8 kJ/m^2^ was attained by manipulating the equivalent ratio of NCO groups in LDI to COOH groups in HDA, while tensile strength (TS) and modulus of the PLA blends were well retained [41]. In the subsequent study, three different types of diisocyanates, namely hexamethylene diisocyanate (HDI), 4,4-diphenylmethane diisocyanate (MDI), and dicyclohexylmethane 4,4′-diisocyanate (HMDI), were further compared to induce dynamic vulcanization of HDA in an attempt to elucidate the critical role of diisocyanate molecular structure in toughening PLA. It was revealed that the reactivity of diisocyanates with HDA contributed to the improvement in interfacial compatibility, while the rigidity of diisocyanates adversely affected the impact toughness of resulting PLA blends. Highly reactive hexamethylene diisocyanate (HDI) with a flexible backbone achieved the optimum toughening effect [42]. In this work, HDA was dynamically vulcanized with LDI or HDI to toughen PLA, which established the feasibility of this strategy and revealed the effect of diisocyanate structure on toughening performance. However, the relationship between the crosslinked network topology of the in situ formed polyamide elastomer and its toughening efficacy has not been addressed yet. The issues mentioned above remain essential to designing high-performance PLA blends via dynamic vulcanization of monomers.

In this study, two carboxylated oleic acids (TCOA and NCOA) were synthesized from technical-grade and high-purity oleic acids, respectively, and then used with hexamethylene diisocyanate (HDI) as toughening monomers to modify PLA via dynamic vulcanization. By tailoring molar ratios of NCO groups to COOH groups between HDI and TCOA (*n*_NCO,HDI_/*n*_COOH,TCOA_), the reaction mechanism, gel content and crosslinking density of the toughening phase, phase morphology, as well as mechanical properties of resulting PLA blends were examined. When replacing TCOA with either NCOA or tetradecanedioic acid (TA) without a dangling chain, the resulting PLA blends were also prepared at the same molar ratio. By comparing TCOA, NCOA, and TA as toughening monomers, the effect of monomer molecular architecture on the cross-linked network topology of the resulting polyamide elastomers and the mechanical properties of the blends was elucidated. This study aims to elucidate how the cross-linked network topology of the toughening phase dominates toughening efficacy in PLA, providing an in-depth understanding of the toughening mechanism at the molecular scale.

## 2. Experimental Section

### 2.1. Materials

Polylactide (PLA 4032D) was obtained from NatureWorks LLC (Plymouth, MN, USA). Hexamethylene diisocyanate (HDI, 99%), Technical-grade oleic acid (TOA, 85%), 2,2-dimethoxy-2-phenylacetophenone (DMPA, 99%), Tetradecanedioic acid (TA), and 3-mercaptopropionic acid (MPA) were purchased from Aladdin Company (Shanghai, China). High-purity oleic acid (NOA, 99%) was procured from Shanghai Titan Technology Co. (Shanghai, China). Ethyl acetate, sodium chloride, trichloromethane, *N,N*-dimethylformamide and Magnesium sulfate anhydrous were supplied by Chuandong Chemical Co., Ltd. (Chongqing, China).

### 2.2. Preparation of Carboxylated Oleic Acids

**Figure 1:**
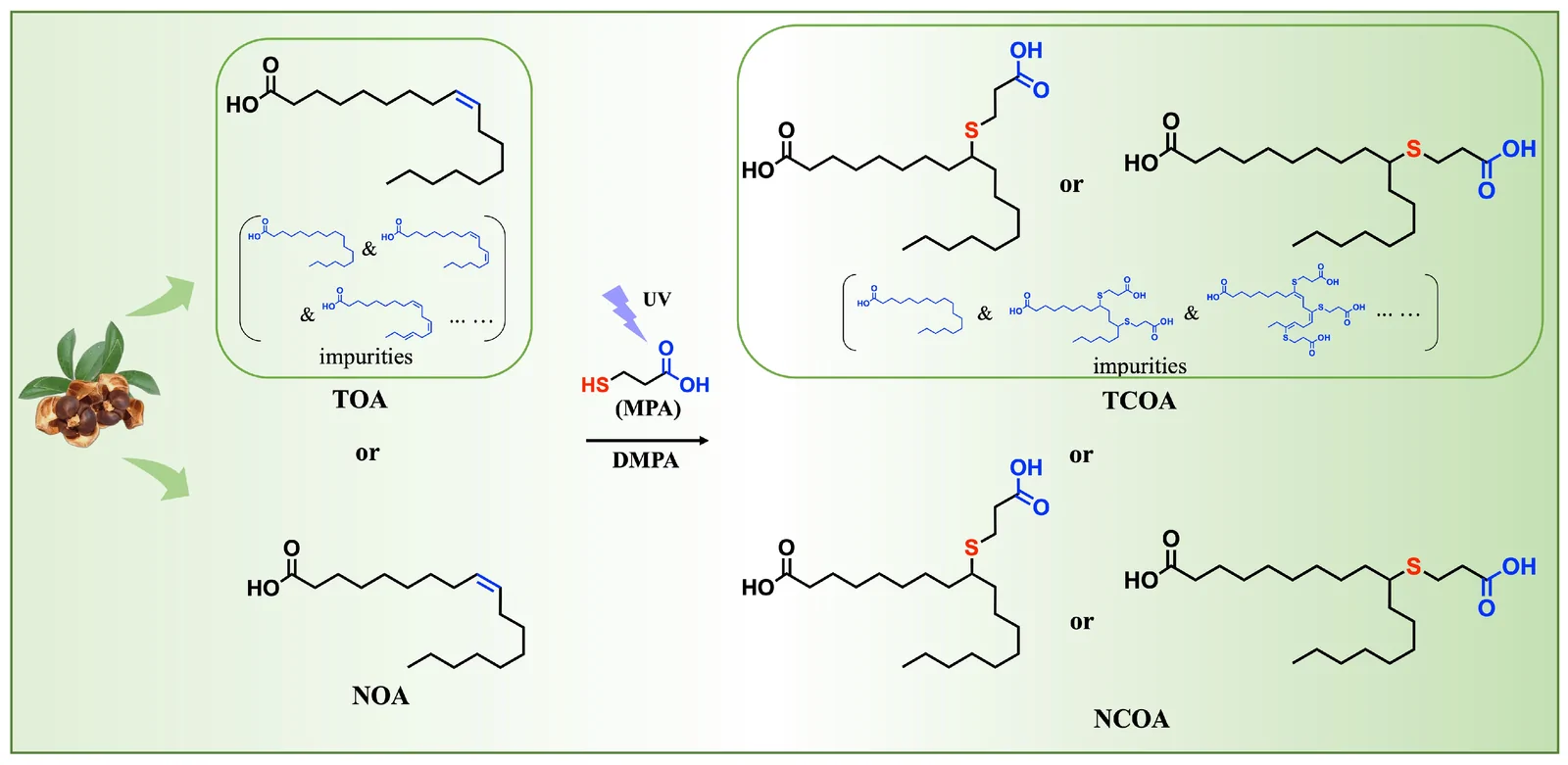
Schematic of the synthesis of TCOA or NCOA by the “thiol−ene” click reaction.

Technical-grade carboxylated oleic acid (TCOA) and high-purity carboxylated oleic acid (NCOA) were prepared via a thiol-ene click reaction in the absence of solvents, employing 85% purity oleic acid (TOA) and high-purity oleic acid (NOA) as the feedstocks, respectively (Figure 1). Taking the preparation of TCOA as an example, TOA (13.29 g, 40 mmol) and 2,2-dimethoxy-2-phenylacetophenone (DMPA, 0.31 g, 3.0 mol% relative to OA) were charged into a 100 mL quartz tube. The mixture was stirred for 5 min in the dark in a chamber equipped with a 50 W UV lamp before rapidly adding MPA (ene-to-thiol molar ratio of 1:1.3). This mixture was subjected to UV irradiation (365 nm) for 3 h under magnetic stirring. Upon reaction completion, the obtained viscous liquid was dissolved in 100 mL of ethyl acetate and washed multiple times with saturated sodium chloride solution to eliminate residual MPA and any disulfide byproducts. After drying over anhydrous MgSO_4_, filtration, and concentration under reduced pressure, the target compound (TCOA) was obtained as a light-yellow viscous liquid as a mixture of regioisomers (yield: 87%). NCOA was prepared similarly, with NOA used instead of TOA (yield: 88%).

^1^H NMR (400 MHz, DMSO-d_6_, δ, ppm): 0.86 (t, ―C***H***_3_), 2.18 (t, ―C***H***_2_―C(O)―, oleic acid chain), 2.45(t, ―C***H***_2_―C(O)―, MPA chain), 2.58–2.64 (m, ―C***H***_2_―S― & ―C***H***―S―), 12.10 (s, ―COO***H***). ^13^C NMR (100 MHz, DMSO-d_6_, 298 K): 174.9 (―***C***OOH, oleic acid chain), 173.7 (―***C***OOH, MPA chain), 45.6 (―***C***H―S―), 14.4 (―***C***H_3_). FT-IR (ATR; cm^−1^): 2922 (CH_2_ aliphatic), 1705 (C=O acid).

### 2.3. Preparation of PLA Blends

PLA pellets were dried in an oven at 75 °C for at least 12 h prior to melt-compounding. Melt compounding was carried out using an XSS-300 torque rheometer (Shanghai Kechuang Rubber & Plastic Equipment Co., Ltd., Shanghai, China) equipped with two counter-rotating rotors. To investigate how the gel content of the toughening phase affected the phase morphology and properties of PLA blends, the total feed of TCOA and HDI in the blends was maintained at 30 wt% for the PLA/TCOA/HDI system. Five different PLA blends were prepared by changing the equivalence ratios of NCO groups in HDI to COOH groups in TCOA (i.e., *n*_NCO,HDI_/*n*_COOH,TCOA_ ranged from 1.2:1 to 2.0:1). These blends are denoted as PLA/COPA-1.2, PLA/COPA-1.4, PLA/COPA-1.6, PLA/COPA-1.8, and PLA/COPA-2.0, respectively.

A designated amount of pre-dried PLA pellets was first charged into the rheometer chamber set to 170 °C and a rotor speed of 60 rpm for approximately 3 min to ensure complete melting of the PLA. Subsequently, TCOA was added by an injector and then continuously mixed with the PLA matrix for another 2 min. Upon adding HDI to induce dynamic vulcanization, the rotor speed was immediately increased to 100 rpm to ensure a complete reaction. Once the torque stabilized, the reaction was terminated, and the material was quickly removed from the chamber and cut into small granules.

PLA/NCOA/HDI and PLA/TA/HDI systems were prepared using a similar procedure to examine how the molecular architecture of the toughening monomers affected the morphology and mechanical properties of PLA blends. This was achieved by fixing the n_NCO,HDI_/n_COOH, carboxylated fatty acids_ molar ratio at 1.8:1. The total feed of difunctional fatty acids and HDI in the blends was maintained at 30 wt%, and TCOA was replaced with either NCOA or tetradecanedioic acid (TA). These systems were designated as PLA/NCOPA-1.8 and PLA/TAPA-1.8, respectively. For comparison, both neat PLA and a PLA/TCOA control sample were prepared under identical conditions, with the latter containing the same TCOA content (based on total mixture mass) as the PLA/COPA-1.8 blend and designated as “PLA/TCOA”. All samples were hot-pressed at 180 °C and 20 MPa to produce sheets with thicknesses of 1 mm and 3 mm for subsequent tensile tests and notched impact tests, respectively.

### 2.4. Nuclear Magnetic Resonance Spectroscopy

^1^H and ^13^C nuclear magnetic resonance (NMR) spectra were recorded with a Bruker NMR spectrometer (Bruker, Rheinstetten, Germany), employing dimethyl sulfoxide (DMSO-d_6_) as the solvent. Solid-state ^13^C NMR spectroscopy of the chloroform-insoluble residue of the PLA/COPA-1.8 blend was performed on a Bruker 600 M spectrometer (Bruker, Rheinstetten, Germany).

### 2.5. Fourier Transform Infrared (FTIR) Spectroscopy

FTIR spectra of PLA blends were recorded using a Nicolet is50 spectrophotometer (Thermo Fisher Scientific, Waltham, MA, USA) over the wavenumber range of 4000 to 400 cm^−1^, at a resolution of 4 cm^−1^ with 32 scans.

### 2.6. Determination of Gel Content

A pre-weighed PLA blend (*W*_1_) was wrapped in filter paper (*W*_0_) and subjected to Soxhlet extraction with excess chloroform (CHCl_3_) for 48 h. The filter paper with the insoluble residue was recovered and dried at 70 °C to constant weight, recorded as *W*_2_. The gel content in the blends was determined using the following equation:

(1) $$\mathrm{Gel}\;\mathrm{content}\;=\;\frac{W_{2}-W_{0}}{W_{1}}\times 100\%$$

### 2.7. Determination of Apparent Crosslinking Density

The apparent crosslink density of the crosslinked toughening phase in PLA blends was quantitatively assessed via swelling tests according to established procedures [43]. An oven-dried insoluble residue (*m*_1_) obtained from CHCl_3_ extraction of the PLA blend was immersed in *N,N*-dimethylformamide (DMF) at room temperature for 3 days to achieve swelling equilibrium. Upon removal, the insoluble product was carefully wiped with filter paper to remove surface solvent and then reweighed (*m*_2_). The volume fraction (*V*_r_) of the swollen crosslinked phase in DMF, calculated using the equation below, was taken as a measure of its apparent crosslink density [44].

(2) $$V_{r}=\frac{1}{1+\left(\frac{m_{2}}{m_{1}}-1\right)\;\frac{\rho_{r}}{\rho_{s}}}$$

where *ρ*_s_ and *ρ*_r_ denote the densities of DMF and the crosslinking toughening phase, assuming values of 0.94 and 1.15 g/cm^3^, respectively.

### 2.8. Thermogravimetric Analysis (TGA)

Thermogravimetric analysis (TGA) was conducted on a TGA Q50 (NETZSCH Instruments, Selb, Germany) under a nitrogen atmosphere at a heating rate of 10 °C/min over a temperature range from room temperature to 700 °C.

### 2.9. Mechanical Testing

Tensile testing was performed at room temperature on an ED26.104 universal testing machine (i-test equipment Co., Ningbo, China) at a crosshead speed of 5 mm/min according to ASTM D638-03 [45]. Dumbbell-shaped specimens with a width of 4 mm and a gauge length of 25 mm were prepared from 1 mm-thick compression-molded sheets. Notched Izod impact strength was measured using a DWS-6069 Pendulum Impact Tester (Jiangsu Davos Machinery Technology Co., Yangzhou, China) at room temperature according to ASTM D256-05 [46]. The specimens had dimensions of 63.5 mm × 12.5 mm × 3.2 mm, with a notch depth of 2.5 mm. For each sample, at least five specimens were tested, and the results reported are the average values.

### 2.10. Dynamic Mechanical Analysis (DMA)

The DMA 850 (TA Instruments, New Castle, DE, USA) was employed for dynamic mechanical analysis (DMA) of PLA blends in single-cantilever mode with an oscillation frequency of 1 Hz. The scanning temperature range was from −100 °C to 150 °C with a scanning speed of 3 °C/min.

### 2.11. Dynamic Rheological Measurement

Dynamic rheological measurements were conducted using an Anton Paar MCR 302 rotational rheometer (Anton Paar, Graz, Austria) equipped with a 25 mm parallel-plate geometry under small-amplitude oscillatory mode. Disk-shaped specimens with a diameter of 25 mm and a thickness of 1 mm were tested at 185 °C. All frequency-sweep measurements were performed within the linear viscoelastic region.

### 2.12. Scanning Electron Microscopy (SEM)

The Hitachi Regulus 8100 (Hitachi, Tokyo, Japan) scanning electron microscope (SEM) was employed to examine the phase morphology of PLA blends. Test specimens were prepared by cryogenic fracture in liquid nitrogen, and the fracture surfaces were subsequently sputter-coated with gold.

### 2.13. Transmission Electron Microscopy (TEM)

The phase morphology of PLA blends was investigated using a JEM-1230 (JEOL Ltd., Tokyo, Japan) transmission electron microscope (TEM). Cryo-ultrathin sections of the PLA blend samples were obtained using an EM UC7 microtome (Leica, Wetzlar, Germany) and subsequently stained with ruthenium tetraoxide (RuO_4_) vapor. The average particle size and size distribution were estimated only for the PLA/COPA-1.2 blends with a distinct sea-island morphology, as the individual particle contours in the other blends were difficult to clearly identify owing to their highly irregular and interconnected shapes. Particle analysis was performed on at least six independent TEM images using ImageJ software (version 1.54r), and the weight-average particle diameter (*d*_w_) was then calculated with the following equation [34]:

(3) $$d_{w}\;=\;\frac{\sum n_{i}{d_{i}}^{2}}{\sum n_{i}d_{i}}$$

where *n*_i_ is the number of particles with the particle diameter *d*_i_.

### 2.14. Determination of Acid Value

The acid value, as defined in ASTM D1980-87 [47], is the number of milligrams of potassium hydroxide (KOH) required to neutralize the fatty acids in 1 g of sample. The determination is carried out by dissolving the sample in hot ethyl alcohol and titrating with a standardized 0.5 N KOH (or NaOH) solution using phenolphthalein as an indicator. The acid value is calculated as follows:

Acid value = (V × N × 56.1)/S(4)

where V is the volume of KOH or NaOH solution used for titration (mL), N is the normality of the KOH or NaOH solution, and S is the sample weight (g).

### 2.15. Differential Scanning Calorimetry (DSC)

Differential scanning calorimetry (DSC) was carried out using a DSC Q2000 (TA Instruments, USA) under N_2_ at a heating rate of 10 °C/min. Based on the first heating cycle, the relative crystallinity of the PLA component in the blends was estimated according to the equation below:

(5) $$X_{c}=\frac{{\Delta H}_{m}-{\Delta H}_{cc}}{\omega_{f,PLA}\times {\Delta H}_{m}^{0}}\times 100\%$$

where Δ$H_{m}^{0}$ denotes the melting enthalpy of 100% crystalline PLA (93.7 J/g), ω_f,PLA_ is the weight fraction of the PLA component in the blends, Δ*H*_cc_ and Δ*H*_m_ correspond to the cold crystallization and melting enthalpies in the first heating cycle, respectively.

## 3. Results and Discussion

### 3.1. Chemical Characterization of TCOA and NCOA

**Figure 2:**
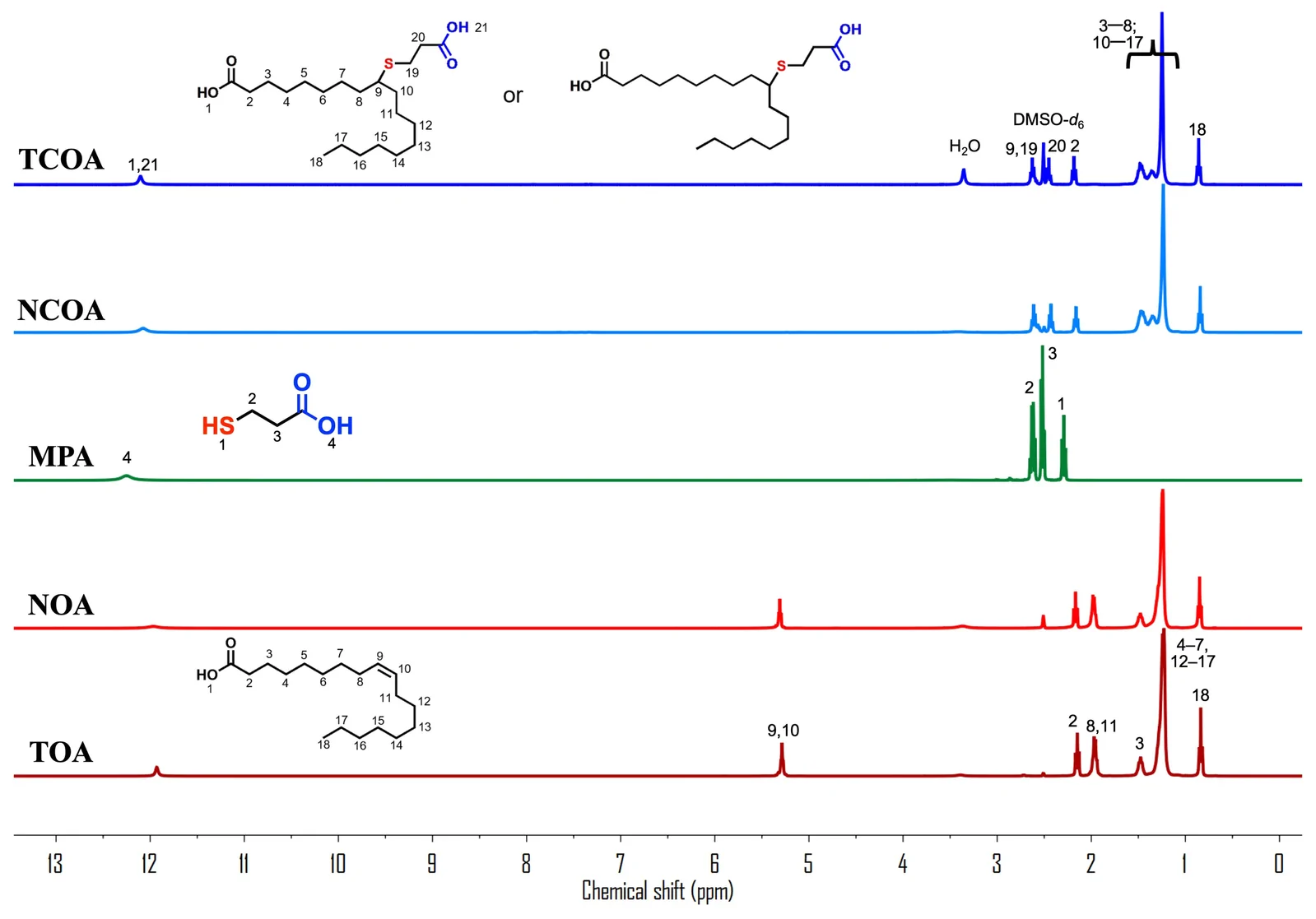
^1^H NMR spectra of TOA, NOA, MPA, NCOA, and TCOA.

Carboxylated fatty acids (TCOA and NCOA) were synthesized using TOA and NOA as starting materials via an environmentally friendly, solvent-free thiol-ene click reaction. The chemical structures of TCOA and NCOA were confirmed by ^1^H NMR, ^13^C NMR, and FTIR. Figure 2 represents the ^1^H NMR spectra of TOA, NOA, MPA, NCOA, and TCOA. The ^1^H NMR spectra of TOA and COA displayed a characteristic peak at 5.29 ppm, attributed to the protons in the carbon-carbon double bonds (―C***H***=C***H***―). In the spectrum of MPA, the characteristic peaks corresponding to the thiol (―S***H***) and carboxylic acid groups (―COO***H***) were observed at 2.29 and 12.25 ppm, respectively. Compared to the spectra of TOA and NOA, the signals corresponding to ―C*H*=C*H*― bonds were undetectable in TCOA and NCOA, confirming the complete conversion of the olefinic feedstock. The spectrum of the products further displayed the characteristic carboxyl peak at approximately 12.1 ppm. Additionally, the signals observed in the range of 2.58–2.64 ppm were attributed to the protons of thioether products (―C***H***_2_―S―) and (―C***H***―S―).

**Figure 3:**
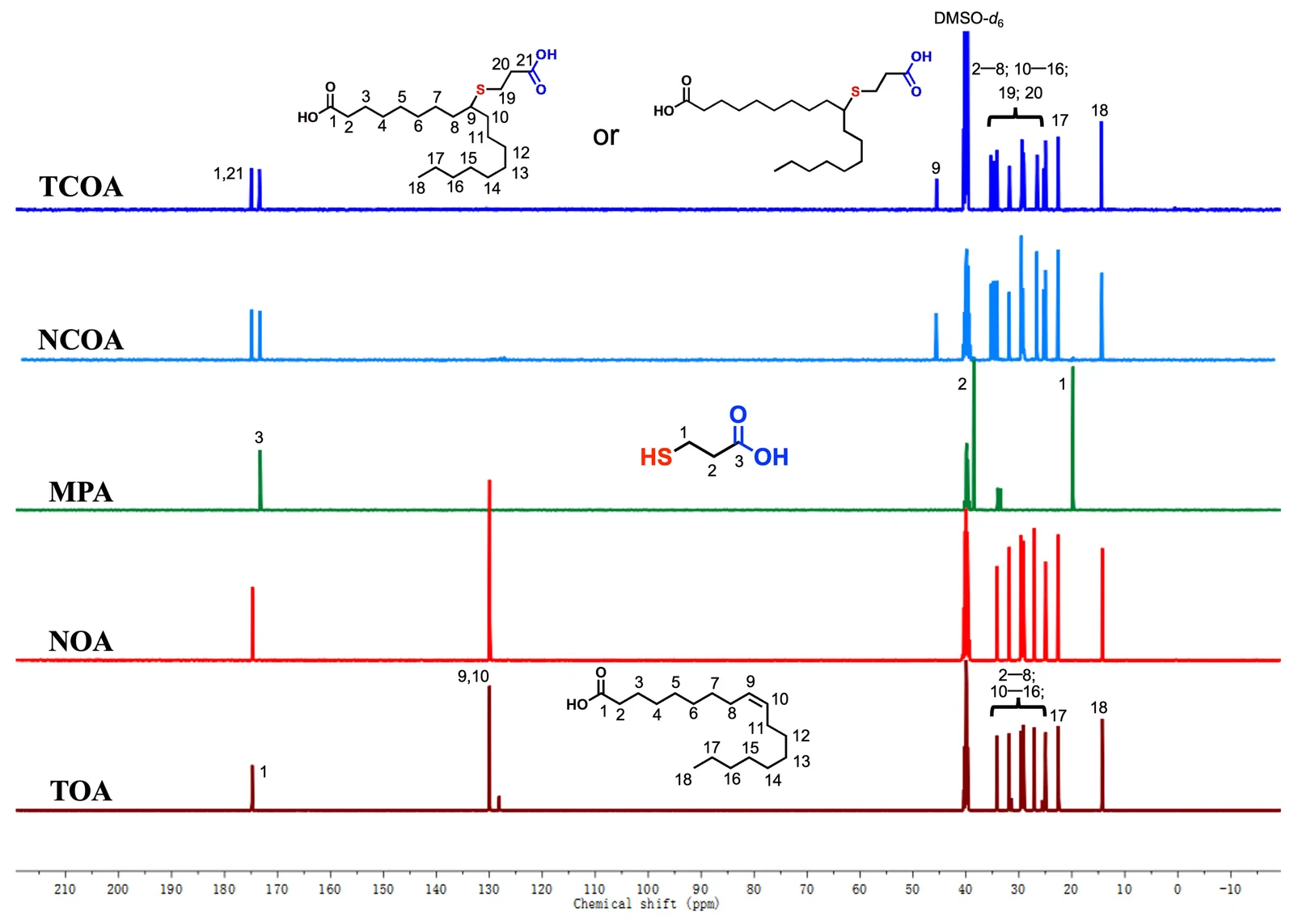
^13^C NMR spectra of TOA, NOA, MPA, NCOA and TCOA.

As shown in the ^13^C NMR spectra (Figure 3), the peak at 130.0 ppm derived from carbon atoms in the double bond of the oleic acid chain disappears for both TCOA and NCOA monomers. Correspondingly, new peaks appeared at 173.4 ppm (―***C***OOH) and 45.6 ppm (―***C***H―S―). The FTIR spectra of TOA, NOA, MPA, NCOA, and TCOA were presented in Figure S1. In the spectra of TCOA and NCOA, the absorption band at 3005 cm^−1^, corresponding to the C―H stretching vibration of the double bond (―CH=CH―), was no longer observed. Concurrently, the band at 1705 cm^−1^, associated with the C=O stretching of the carboxylic acid, exhibited increased intensity. These results confirm the successful incorporation of MPA via thiol-ene click reaction.

To determine the exact amount of carboxylic groups in the reactive monomers for PLA dynamic vulcanization, the acid values of TCOA and NCOA were measured according to ASTM D1980-87, yielding results of 289.5 ± 1.2 and 291.4 ± 6.2 KOH/g, respectively. Furthermore, the initial decomposition temperatures (*T*_5%_) of TCOA and NCOA were determined by thermogravimetric analysis (TGA) to be 266 °C and 256 °C, respectively, as shown in Figure S2. These values are markedly higher than the melt-compounding temperature (170 °C) used in this work, confirming that both monomers maintain thermal stability during the entire dynamic vulcanization process.

### 3.2. PLA/COPA Blends Prepared with Different NCO/COOH Molar Ratios

#### 3.2.1. Reaction Mechanism of PLA/COPA Blends

PLA/COPA blends were fabricated using TCOA as the toughening monomer through HDI-induced dynamic vulcanization. The dynamic vulcanization process was monitored by measuring the torque of the samples (Figure S3). A significant reduction in torque was immediately observed after adding TCOA to the blending chamber due to its lubricating effect. Following the addition of HDI, the melt torque initially increased upon extending the mixing time and then gradually leveled off, indicating a reaction between TCOA and HDI. In addition, a large amount of gas was released from the mixing chamber during melt blending. This was likely caused by CO_2_ produced through the reaction between –COOH and –NCO groups. The evolved CO_2_ escapes from the mixing chamber through the clearances around the rotor shafts, and the subsequent hot-pressing at 180 °C and 20 MPa further eliminates residual gas.

**Figure 4:**
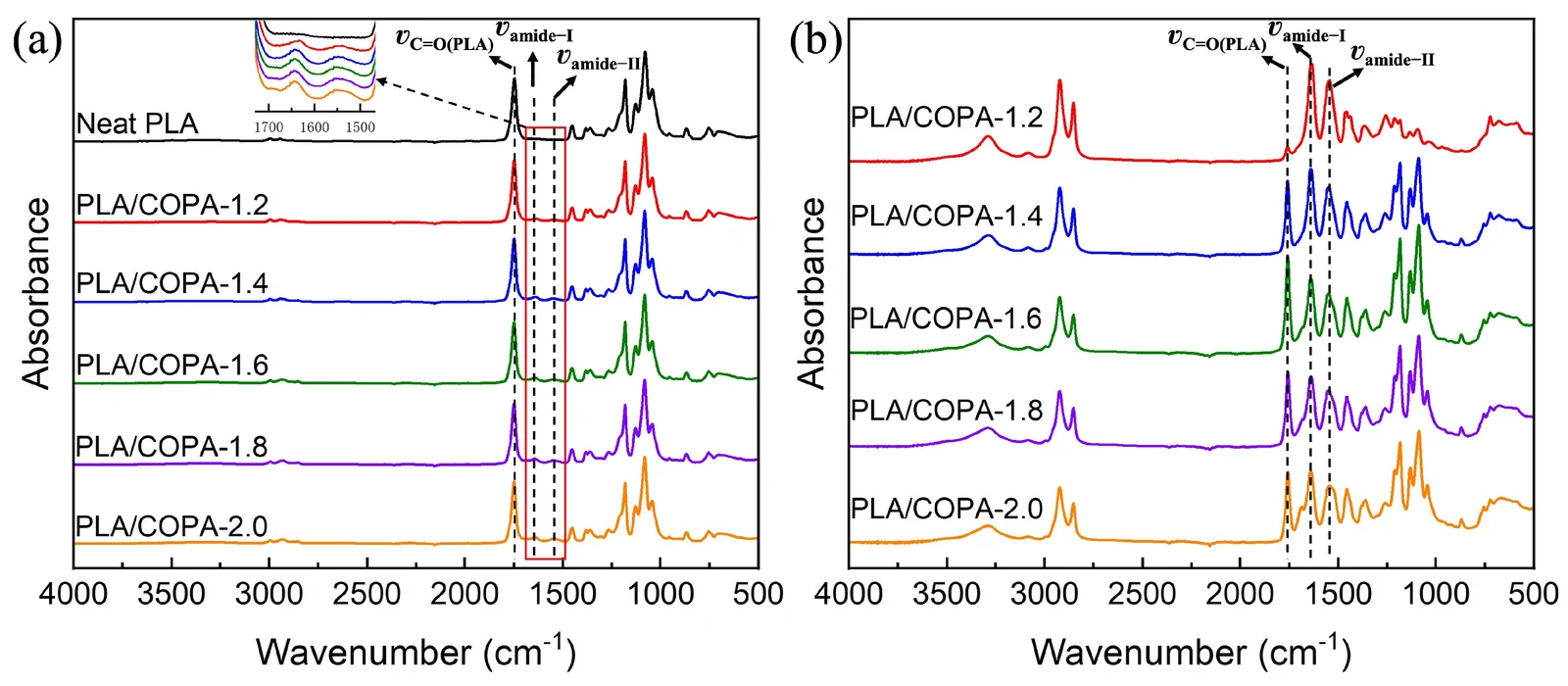
FTIR spectra of neat PLA and PLA/COPA blends prepared at different *n*_NCO,HDI_/*n*_COOH,TCOA_ ratios: (**a**) non-extracted and (**b**) the CHCl_3_-etching treatment.

The FTIR spectra of neat PLA and non-extracted PLA/COPA blends prepared at different *n*_NCO,HDI_/*n*_COOH,TCOA_ ratios are shown in Figure 4a. In the spectra of PLA/COPA blends, characteristic peaks assigned to the amide I and amide II bands were observed at 1643 cm^−1^ and 1548 cm^−1^, respectively, indicating that the TCOA in situ reacts with HDI to form polyamide intermediates containing unstable N-carboxanide moieties, which rapidly rearrange to generate amide bonds through the release of CO_2_ [48,49]. Furthermore, the absence of the –NCO absorption band for HDI at 2250 cm^−1^ indicates that HDI was completely consumed during dynamic vulcanization. It should be noted that reactions between isocyanates and carboxylic acids at melt-processing temperatures can proceed through complex pathways, and the resulting network may also contain urea, acylurea, biuret, and allophanate linkages as side products. The FTIR evidence presented here supports the formation of a crosslinked network but cannot definitively distinguish among these specific structural motifs.

To confirm the occurrence of crosslinking reactions, chloroform (CHCl_3_) etching was employed to remove free PLA components, unreacted monomers, and non-crosslinked polyamide products from the PLA/COPA blend [40]. The insoluble residues of the PLA/COPA blends with different composition ratios were analyzed by FTIR spectroscopy (Figure 4b). The characteristic amide-I (1643 cm^−1^) and amide-II (1548 cm^−1^) bands of all insoluble residues were observed in the FTIR spectra, confirming the formation of a cross-linked COPA elastomer. The gel content of the PLA/COPA blend initially increased and then gradually plateaued as *n*_NCO,HDI_/*n*_COOH,TCOA_ increased (Table S1). When the ratio was 1.4 or higher, the gel content consistently remained above 25%. Moreover, the apparent crosslinking density (*V*_r_) of the PLA/COPA blends continued to increase as the ratio was raised. In addition, the stretching vibration of the PLA ester C=O group appeared at 1750 cm^−1^ in these residues, confirming that reactive compatibilization occurred between the terminal hydroxyl or carboxyl groups of PLA and the isocyanate ones of the COPA phase. This compatibilization enabled the generation of PLA-*b*-COPA copolymers, which enhanced interfacial adhesion between the two phases. To quantitatively assess the compatibility between PLA and the polyamide phase, the area ratios of the FTIR absorption peaks at 1758 cm^−1^ and 1643 cm^−1^ (A_C=O,PLA_/A_amide-I_) after thorough CHCl_3_ etching were calculated. The peak area ratio of the PLA/COPA-1.2 blend was 0.22. As the *n*_NCO,HDI_/*n*_COOH,TCOA_ ratio increased, this A_C=O,PLA_/A_amide-I_ value rose from 0.22 to 0.71 and then decreased slightly. This indicates that when the ratio exceeded 1.0:1, the amount of PLA chains grafted onto the crosslinked COPA network increased significantly.

To further confirm the occurrence of interfacial compatibilization, solid-state ^13^C NMR spectroscopy was performed on the chloroform-insoluble residue of the PLA/COPA-1.8 blend (Figure S4). Distinct carbonyl resonance peaks are observed at 174–170 ppm, corresponding to the PLA ester carbonyl (C=O) and polyamide imide carbonyl groups. Most notably, a peak at 159 ppm was attributed to the urethane carbonyl group (–NH–CO–O–), which originated from the reaction between the –NCO groups of HDI and the terminal –OH groups of PLA. This provides direct evidence for the formation of PLA-b-COPA copolymers between the COPA toughening phase and the PLA matrix.

**Figure 5:**
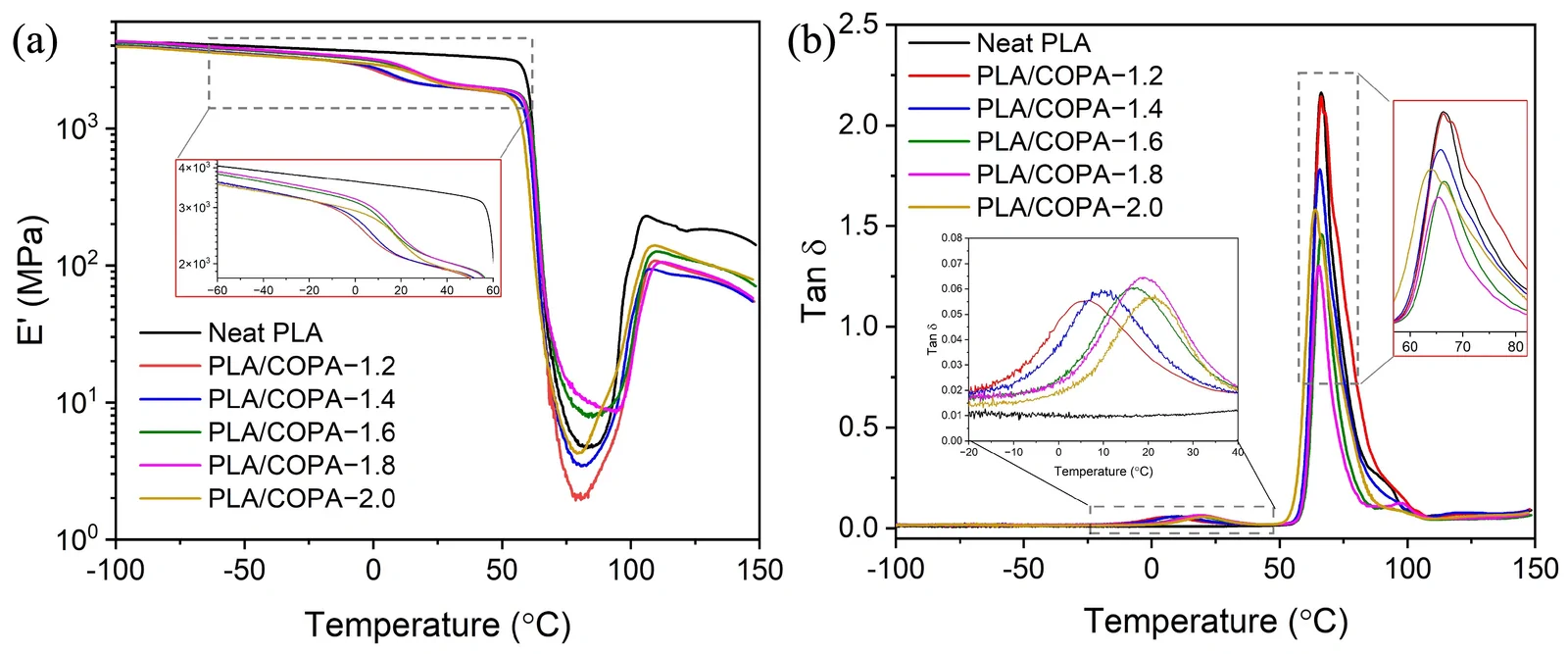
Storage modulus (**a**) and damping factor (**b**) for neat PLA and PLA/COPA blends with different *n*_NCO,HDI_/*n*_COOH,TCOA_ ratios.

Figure 5 presents the storage modulus and damping factor (tanδ) of neat PLA and PLA/COPA blends. The storage modulus curves of all PLA/COPA blends exhibited two distinct decreases, corresponding to glass transition temperatures (*T*_g_) of the COPA and the PLA phases, respectively (Figure 5a). The storage moduli of the PLA/COPA blends were lower than those of neat PLA over a given temperature range (−60 °C to 60 °C), due to the presence of a soft COPA phase formed by in situ polymerization. The temperature dependence of tanδ in the PLA/COPA ones displayed two distinct relaxation peaks, corresponding to the COPA phase and PLA phase, respectively (Figure 5b). For the PLA/COPA-1.2 blend, *T*_g,COPA_ was approximately 5.8 °C. With the *n*_NCO,HDI_/*n*_COOH,TCOA_ ratio becoming 1.4:1, the *T*_g,COPA_ value of the PLA/COPA-1.4 one increased to 9.8 °C, which was approximately 4 °C higher than that of the PLA/COPA-1.2 one. A consistent increase in *T*_g,COPA_ was observed upon further increasing this ratio. When the ratio reached 2.0:1, *T*_g,COPA_ increased to 20.6 °C for the PLA/COPA-2.0 one. This indicates that increasing *n*_NCO,HDI_/*n*_COOH,TCOA_ promoted the formation of a more highly crosslinked COPA phase. Concurrently, the *T*_g,PLA_ values shifted to lower temperatures.

#### 3.2.2. Morphological Evolution of PLA/COPA Blends

**Figure 6:**
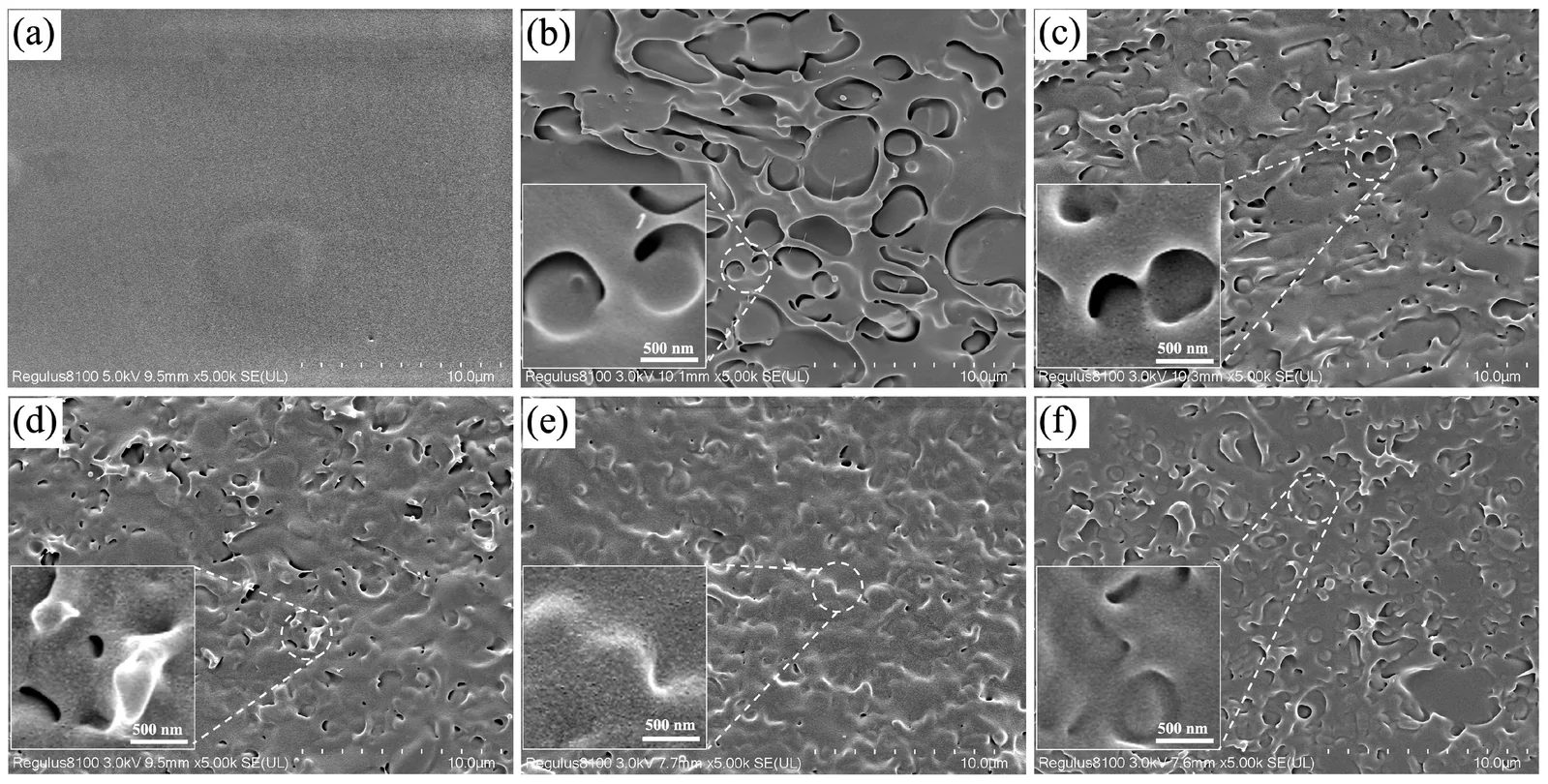
SEM images of cryo-fractured surfaces of neat PLA and PLA blends with different *n*_NCO,HDI_/*n*_COOH,TCOA_ ratios: (**a**) neat PLA; (**b**) PLA/COPA-1.2, (**c**) PLA/COPA-1.4, (**d**) PLA/COPA-1.6, (**e**) PLA/COPA-1.8, and (**f**) PLA/COPA-2.0.

Figure 6 presents SEM micrographs of cryo-fractured surfaces of the neat PLA and PLA/COPA blends with different n_NCO,HDI_/n_COOH,TCOA_ ratios. The cryo-fractured surface of neat PLA was homogeneous, smooth, and devoid of any phase-separated structures (Figure 6a). The PLA/COPA-1.2 blend displayed many discrete particles with a large size dispersion. A large gap was also observed between the two phases, indicating poor interfacial compatibility between the PLA matrix and COPA phases (Figure 6b). Notably, as n_NCO,HDI_/*n*_COOH,TCOA_ increased, the interfacial affinity significantly improved. When the ratio was increased to 1.4:1, the PLA/COPA-1.4 one exhibited finer particles, but phase separation was still observed (Figure 6c). Especially at a ratio of 1.8:1 (Figure 6e), the fractured surface became more homogeneous, with few distinct particles, indicating significantly improved interface compatibility. When the ratio was further increased to 2.0, however, the surface became rougher with discernible particles, indicating that interfacial compatibility began to decline.

**Figure 7:**
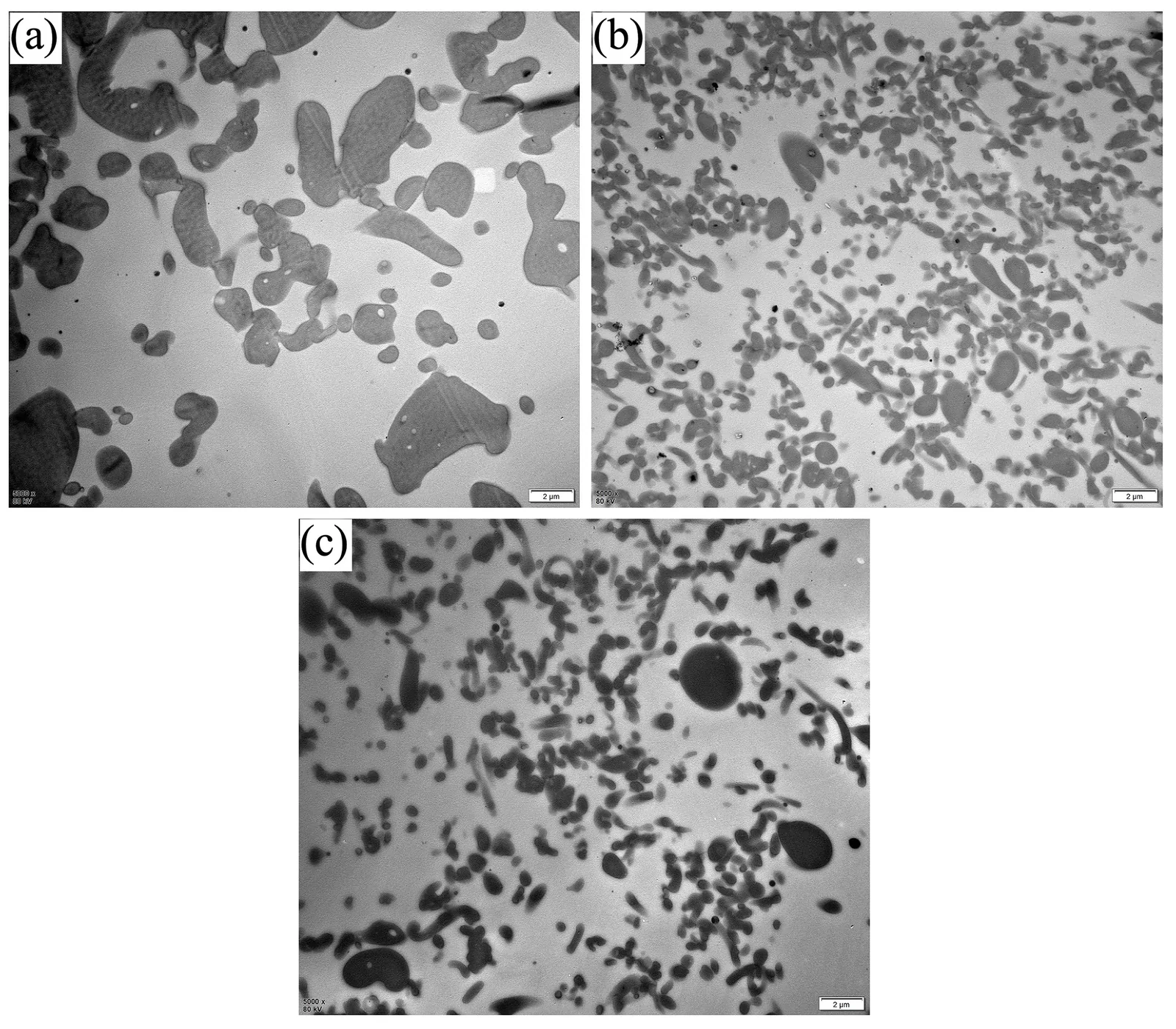
RuO_4_-stained TEM micrographs of PLA blends with different *n*_NCO,HDI_/*n*_COOH,TCOA_ ratios: (**a**) PLA/COPA-1.2, (**b**) PLA/COPA-1.8, (**c**) PLA/COPA-2.0.

To analyze the detailed phase structure of the PLA/COPA blends, RuO_4_-stained ultra-thin cryosection samples were obtained using transmission electron microscopy (TEM). Figure 7 presents TEM images of RuO_4_-stained PLA/COPA-1.2, PLA/COPA-1.8, and PLA/COPA-2.0 blends. A typical “sea-island” phase-separated structure was visible in the PLA/COPA-1.2 blend, which was characterized by irregular, dark COPA particles randomly dispersed throughout the PLA matrix. The *d*_w_ of dispersed COPA particles was estimated to be 1.20 μm, and the particle size distribution was presented in Figure S5. Conversely, pronounced aggregation, with some COPA particles forming highly irregular pseudo-network structures, was observed in both PLA/COPA-1.8 and PLA/COPA-2.0 blends. The COPA domains of the PLA/COPA-1.8 blend exhibited enhanced percolation, indicating the formation of a continuous network.

**Figure 8:**
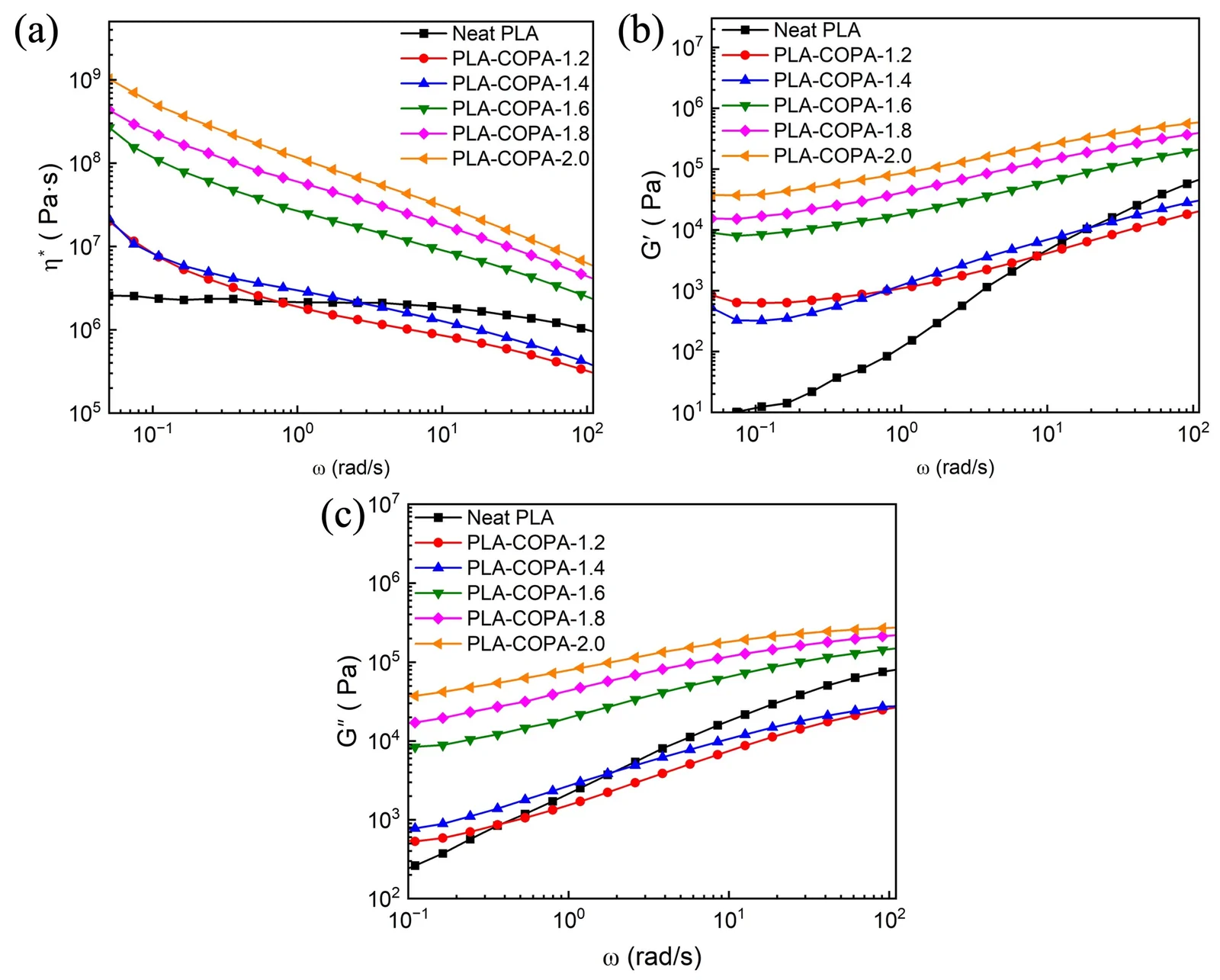
Dynamic rheological properties versus angular frequency at 185 °C for neat PLA and PLA/COPA blends with different *n*_NCO,HDI_/*n*_COOH,TCOA_ ratios: (**a**) complex viscosity (η*); (**b**) storage modulus (G′); (**c**) loss modulus (G″).

To further explore phase morphologies of the PLA/COPA blends, dynamic frequency sweep tests were conducted in small-amplitude oscillatory shear mode. Figure 8a shows the complex viscosity (η*) versus angular frequency (ω) for PLA/COPA blends with different *n*_NCO,HDI_/*n*_COOH,TCOA_ ratios. Neat PLA exhibited typical Newtonian fluid behavior across the entire frequency range. In contrast to neat PLA, the PLA/COPA blends displayed pronounced shear-thinning behavior. When the *n*_NCO,HDI_/*n*_COOH,TCOA_ ratio was below 1.6:1, the complex viscosity (η*) of the resulting PLA/COPA blends was nearly comparable at low frequencies, but significantly higher than that of neat PLA. In contrast, the η* values of the PLA/COPA blends were lower than those of neat PLA at high frequencies. When this ratio was elevated to 1.6:1, the η* value increased significantly by nearly one order of magnitude across the entire frequency range. Moreover, further increases in the ratio led to a continuous increase in the η* of the PLA/COPA blend. Both the storage modulus (G′) and loss modulus (G″) as functions of ω exhibited a strong dependence on *n*_NCO,HDI_/*n*_COOH,TCOA_ (Figure 8b,c). For neat PLA, the rheological behavior exhibited terminal flow characteristics of viscoelastic liquids, where approximately G′∝ω^2^ and G″∝ω. As *n*_NCO,HDI_/*n*_COOH,TCOA_ increased, the G′ and G″ values also increased in the low-frequency region. The terminal slope exhibited a weaker dependence on ω, indicating more pronounced solid-like behavior. This suggests that a crosslinked or entangled COPA network was formed in situ within the PLA matrix by dynamic vulcanization. Further support for this conclusion was provided by the Cole–Cole plot shown in Figure S6b. The Cole-Cole curve for neat PLA showed a single arc. At low ratios of 1.2 or 1.4, the PLA/COPA blend exhibited an arc-shaped profile with a linear tail in the Cole–Cole plot, which was attributed to the formation of a sea-island structure [50]. As the ratio was increased further, only a linear profile was observed in the Cole–Cole plot, a characteristic feature of its unique co-continuous or network-like morphology. Figure S6a presents the Han plots, where the diagonal line represents the isomodal line (G′ = G″). At ratios below 1.6, the Han curves of the PLA/COPA blends remained almost entirely below the isomodal line over the entire frequency range, with G′ consistently lower than G″. This result suggests that the blends still exhibited predominantly viscous behavior in the molten state. At ratios of 1.6:1 or above, however, the Han plot terminal region shifted to lie above the isomodal line (G′ = G″), with G′ and G″ nearly equal at low frequencies. This suggests that elastic response became predominant under these conditions.

To verify the structural stability of the PLA/COPA blends during frequency-sweep measurements at 185 °C, we conducted an isothermal time-sweep test on the PLA/COPA-1.8 blend at a frequency of 1 rad/s for 30 min. The time-sweep data show no monotonic increase in η* over the 30 min duration, confirming that no post-crosslinking occurs during testing (Figure S7). It is worth noting that η* decreases by only about 5% during the first 9 min. Since the frequency sweep (0.05–200 rad/s) requires only 440 s to complete, the changes within this window are negligible and do not affect the interpretation of the frequency sweep curves.

#### 3.2.3. Mechanical Properties of PLA/COPA Blends

**Figure 9:**
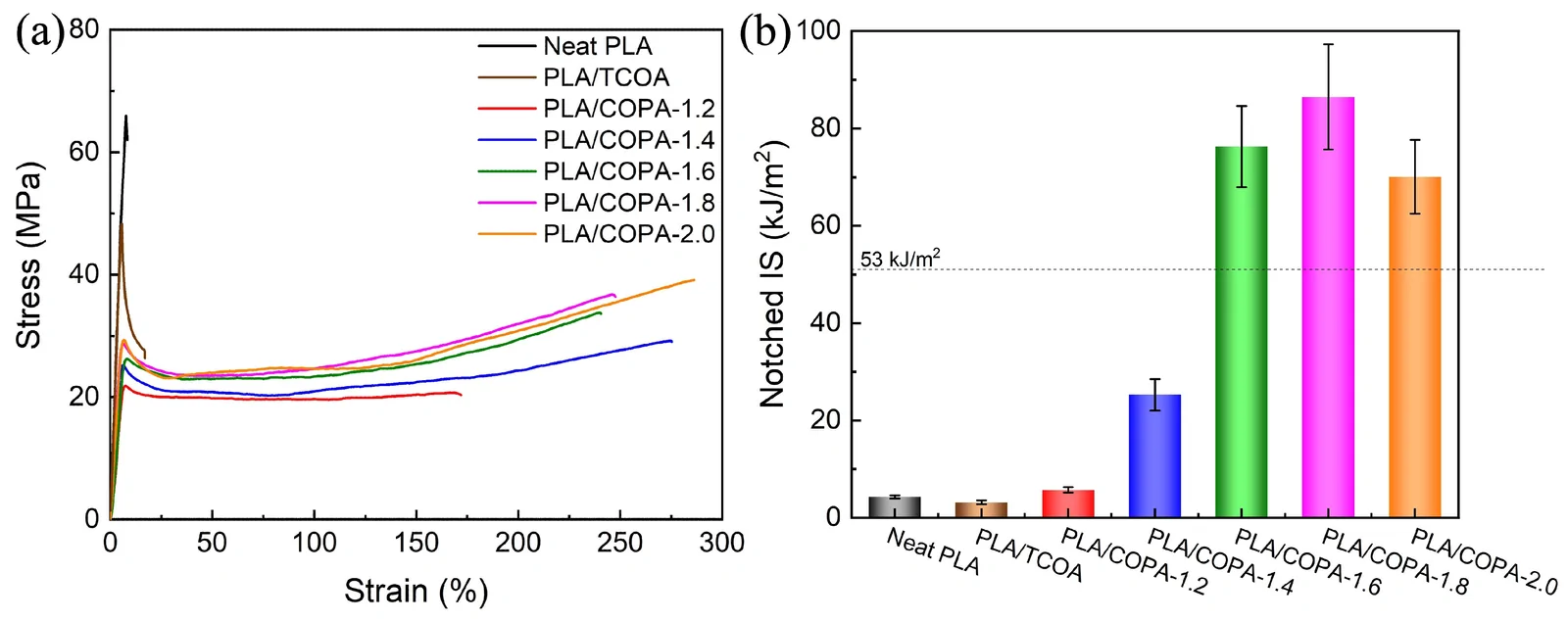
Stress–strain curves (**a**) and column plots of the Izod notched impact strength (IS) (**b**) of the neat PLA and PLA/COPA blends with different *n*_NCO,HDI_/*n*_COOH,TCOA_ ratios.

Figure 9a shows the stress–strain curves of the neat PLA control sample and PLA/COPA blends, and the mechanical properties of the samples are presented in Table S2. Neat PLA exhibited typical brittle fracture behavior, as evidenced by its tensile strength (TS) of 65.7 MPa, elongation at break of 8.5%, and notched impact strength (IS) of only 4.3 kJ/m^2^. In contrast, the PLA/TCOA blend showed a marginal improvement in elongation at break (14.3%) and a similarly low notched IS (3.2 kJ/m^2^), accompanied by a significant reduction in TS (49.8 MPa). These results suggest that TCOA has a limited plasticizing effect and is incapable of effectively toughening PLA. The PLA/COPA blends prepared by HDI-induced dynamic vulcanization demonstrated excellent ductility. When the n_NCO,HDI_/n_COOH,TCOA_ ratio was only 1.2:1, the PLA/COPA-1.2 blend exhibited an elongation at break of 175.1%, which was approximately 20 times higher than that of neat PLA. However, its Young’s modulus and TS decreased significantly to 1.6 GPa and 23.2 MPa, respectively, and strain hardening was not observed. As the *n*_NCO,HDI_/*n*_COOH,TCOA_ ratio further increased, the TS of PLA/COPA samples significantly increased due to strain hardening. Upon increasing *n*_NCO,HDI_/*n*_COOH,TCOA_ to 1.4:1, the TS of PLA/COPA-1.4 reached 28.8 MPa, while its elongation at break further increased to 276.6%, reaching 32.5 times the value of neat PLA. At a ratio of 1.8:1, the TS of PLA/COPA-1.8 one reached 37.0 MPa, presumably due to the increased cross-linking density of the toughening phase. In addition, the reduction in the elongation at break of PLA/COPA samples with higher ratios of 1.6:1 and 1.8:1 may be attributed to a higher cross-linking degree of the toughening phase, which decreased the mobility of its chain segments.

Figure 9b displays the Izod notched IS of neat PLA and the PLA/COPA blends. The PLA/COPA-1.2 blend showed a notched impact strength of merely 5.7 kJ/m^2^, which was not a significant increase compared with neat PLA. This limited toughening was ascribed to poor interfacial compatibility, as confirmed by the morphological features in Figure 6b and Figure 7a. As *n*_NCO,HDI_/*n*_COOH,TCOA_ ratio further increased, the PLA/COPA ones exhibited a brittle-ductile transition with respect to notched IS. As the ratio was further elevated to 1.6:1, the impact toughness increased significantly to 76.3 kJ/m^2^, demonstrating that super-tough PLA materials could be achieved solely by regulating the molar ratio of the two vulcanizing monomers. At an *n*_NCO,HDI_/*n*_COOH,TCOA_ ratio of 1.8:1, the PLA/COPA-1.8 one exhibited the maximum notched IS value of 86.5 kJ/m^2^, which was 20.1 times higher than that of neat PLA. When the ratio was further increased to 2.0:1, the notched IS value of the blend slightly decreased, but remained at least 70 kJ/m^2^.

Figure S10 presents the DSC curves obtained during the first heating scan, and the corresponding data are summarized in Table S3. Neat PLA exhibited a glass transition temperature (*T*_g_) of 60.7 °C. The *T*_g_ values of the PLA component in all blends were almost identical to that of neat PLA, confirming the absence of significant plasticization. Compared with neat PLA, the blends showed *T*_cc_ values ranging from 101.6 to 113.8 °C, indicating altered crystallization ability of the PLA segments. The crystallinity (*X*_c,PLA_) of the PLA component in the blends remained at a relatively low level and did not increase substantially relative to neat PLA. Therefore, PLA crystallization hardly accounts for the markedly increased impact strength (IS) observed in the toughened blends.

Table S4 compares the mechanical properties of the present PLA/COPA blends with representative super-tough PLA systems prepared via dynamic vulcanization. The PLA/COPA-1.8 blend achieved a tensile strength of 37.0 MPa (56% retention relative to neat PLA) and a notched impact strength of 86.5 kJ/m^2^ (20.1-fold enhancement). This level of impact toughening is comparable to that of excellently performing diisocyanate/dicarboxylic acid systems previously reported, including PLA/HDAPA20 (30.5-fold) [41] and PLA/HPA (37.7-fold) [51]. In terms of TS retention, the present system (56%) is similar to PLA/VESO (59%) [36] and PLA/PBSFG (54%) [28], and superior to most peroxide-initiated systems (44–55%) [21,33,52]. These results demonstrate that tailoring the crosslinked network topology of the COPA phase provides an effective route to optimize the stiffness–toughness balance via a simple one-step melt-blending process, without the need for pre-synthesized elastomers or multi-step processing. Other representative systems include PLA toughened by polyurethane elastomer prepolymers [53], PCL/MDI [54], bio-based polyether PO3G/HDI [55], and unsaturated bioelastomer/PDLA ternary blends [56].

Our previous work [40] demonstrated that during dynamic vulcanization of a PLA/dibasic fatty acid/HDI system, HDI first underwent in situ polymerization with an equimolar amount of dibasic fatty acid to form an uncrosslinked polyamide elastomer. This was followed by crosslinking via reactions of the secondary amide groups with excess HDI. The resulting NCO-terminated toughening elastomer reacted with the terminal –OH and –COOH groups of PLA to form PLA-b-polyamide copolymers, thereby achieving interfacial compatibilization. For PLA/COPA blends, increasing the *n*_NCO,HDI_/*n*_COOH,TCOA_ ratio provided excess isocyanate groups that participated in crosslinking with secondary amide groups and compatibilization with PLA terminal –OH/–COOH groups during dynamic vulcanization. This increased the gel content of the toughening phase and progressively enhanced interfacial adhesion.

### 3.3. PLA Blends with Different Molecular Architectures

The toughening monomer TCOA in the PLA/COPA systems was synthesized using TOA (purity ≥ 85%) as a starting material. However, TOA contains minor amounts of impurities such as monofunctional saturated fatty acids (e.g., palmitic acid and stearic acid) and polyunsaturated fatty acids (e.g., linoleic acid and linolenic acid) [57]. Thus, the obtained TCOA contains both mono-functional and multi-functional fatty acids, which may affect the homogeneity of the crosslinked network of the toughening phase, thereby influencing the performance of the PLA blend. Therefore, to investigate the effect of the molecular architecture of toughening monomers, we prepared PLA/NCOPA and PLA/TAPA blends by melt blending, using NOA and TA as the toughening monomers, respectively.

#### 3.3.1. Mechanical Properties

**Figure 10:**
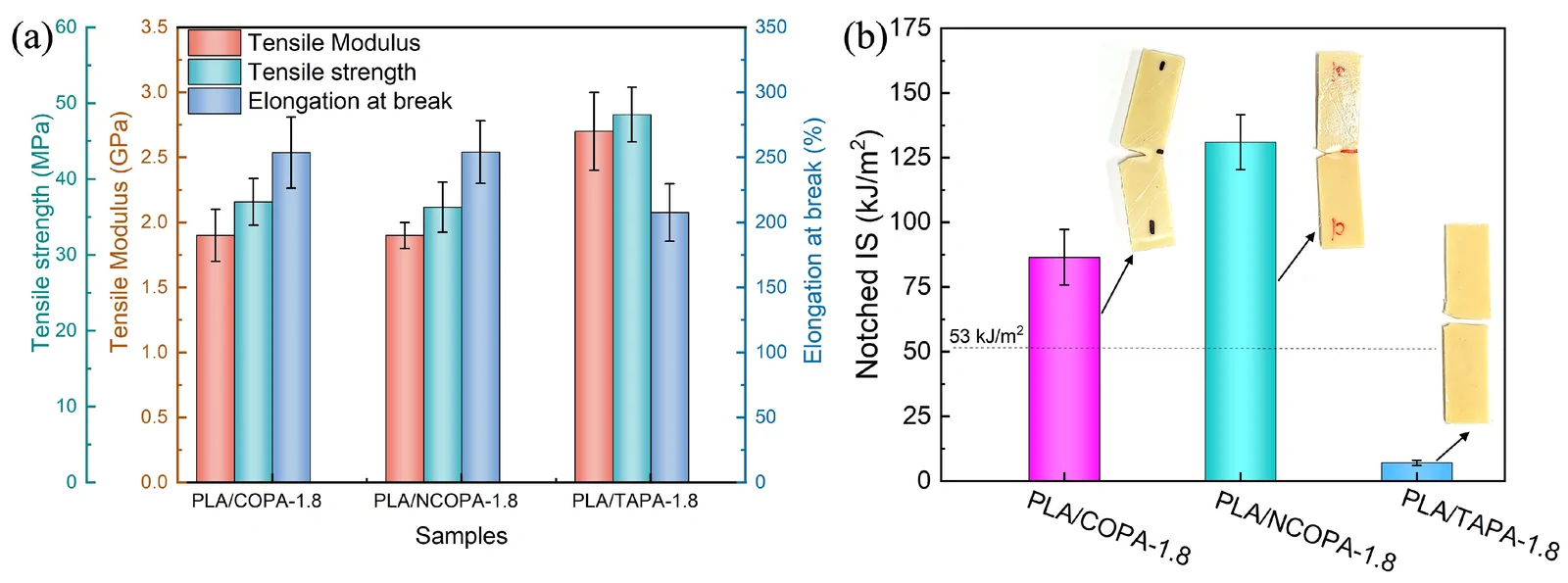
Tensile properties (**a**) and Izod notched impact strength (**b**) of three PLA blends.

The mechanical properties of three PLA blends with different molecular architectures are shown in Figure 10, and the corresponding data are summarized in Table S2. The tensile properties of the PLA/COPA-1.8 blend were comparable to those of the PLA/NCOPA-1.8 blend, which used NCOA as the toughening monomer. However, the notched IS of the former was only about 0.7 times that of the latter (131.0 ± 10.6 kJ/m^2^). This indicates that mono- and multi-functional fatty acid impurities in the toughening monomers significantly affected the impact toughness of the blends. Even under impact loads as high as 5.5J, the notched specimens of both blends showed the non-break fracture mode, and the thickness of the unbroken specimens of the latter appeared to be higher. The energy absorbed by each specimen did not exceed 85% of the pendulum capacity (5.5 J), in accordance with ASTM D256-05. The reported impact strength values are valid measurements, although the actual toughness may be higher as the specimens were not fully fractured. In addition, PLA/TAPA-1.8 prepared using tetradecanedioic acid with the linear backbone exhibited a superior tensile modulus (2.7 GPa) and tensile strength (48.5 MPa) to PLA/COPA-1.8. Despite a slightly lower ductility (207.7%), it maintained a high elongation at break. Notably, the notched IS of PLA/TAPA-1.8 one was the lowest, with a value of only 7.0 ± 1.1 kJ/m^2^. This was likely due to the higher crosslink density of the polyamide elastomer derived from the TA monomer without long alkyl dangling chains. This assumption was corroborated by the subsequent apparent crosslink density and microstructural analyses.

#### 3.3.2. Reaction Mechanism

**Figure 11:**
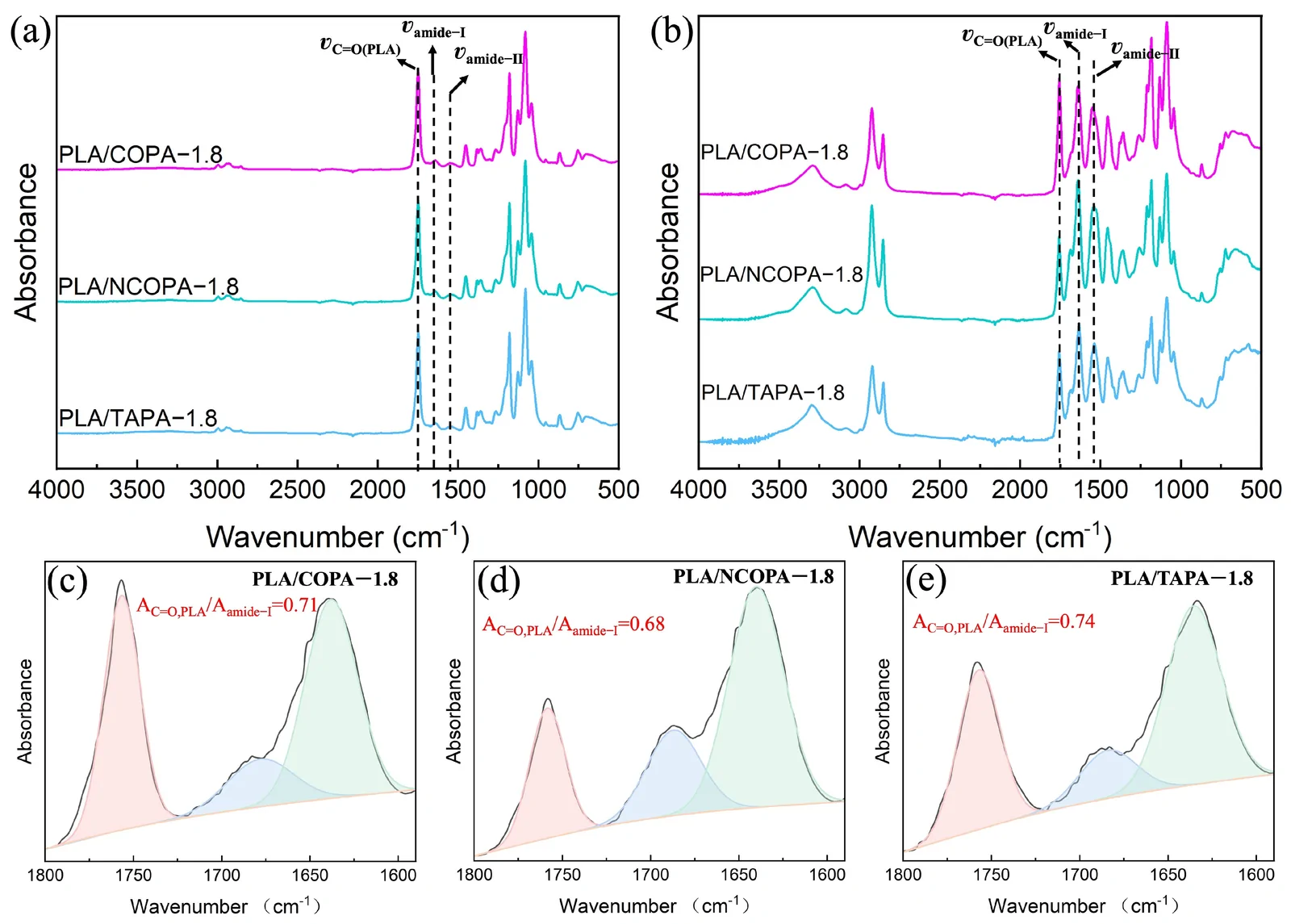
(**a**,**b**) FTIR spectra of PLA blends with different molecular architectures: before (**a**) and after (**b**) the CHCl_3_-etching treatment; (**c**–**e**) analysis of the FTIR peaks in the C=O and amide I regions for the same blends.

Figure 11a presents FTIR spectra of the non-extracted PLA blends with different molecular architectures. The characteristic amide I (1640 cm^−1^) and amide II (1545 cm^−1^) absorption peaks were observed in the spectra of all three samples, confirming that polyamide was formed by the in situ polymerization of long-chain fatty acid dicarboxylic acids with diisocyanates during dynamic vulcanization. After etching with CHCl_3_, the dried insoluble residue was collected and characterized by FTIR spectroscopy (Figure 11b). All three blends exhibited characteristic amide bands at 1640 and 1545 cm^−1^, verifying the formation of crosslinked polyamide elastomers. The three PLA blends exhibited similar gel contents, with values of 28.5%, 27.0%, and 28.51.6% for PLA/COPA-1.8, PLA/NCOPA-1.8, and PLA/TAPA-1.8, respectively. Furthermore, the apparent crosslinking densities of the PLA/COPA-1.8 and PLA/NCOPA-1.8 blends were determined to be 0.52 and 0.58, respectively, whereas the PLA/TAPA-1.8 blend exhibited a notably higher density of 0.65 (Table S1).

**Figure 12:**
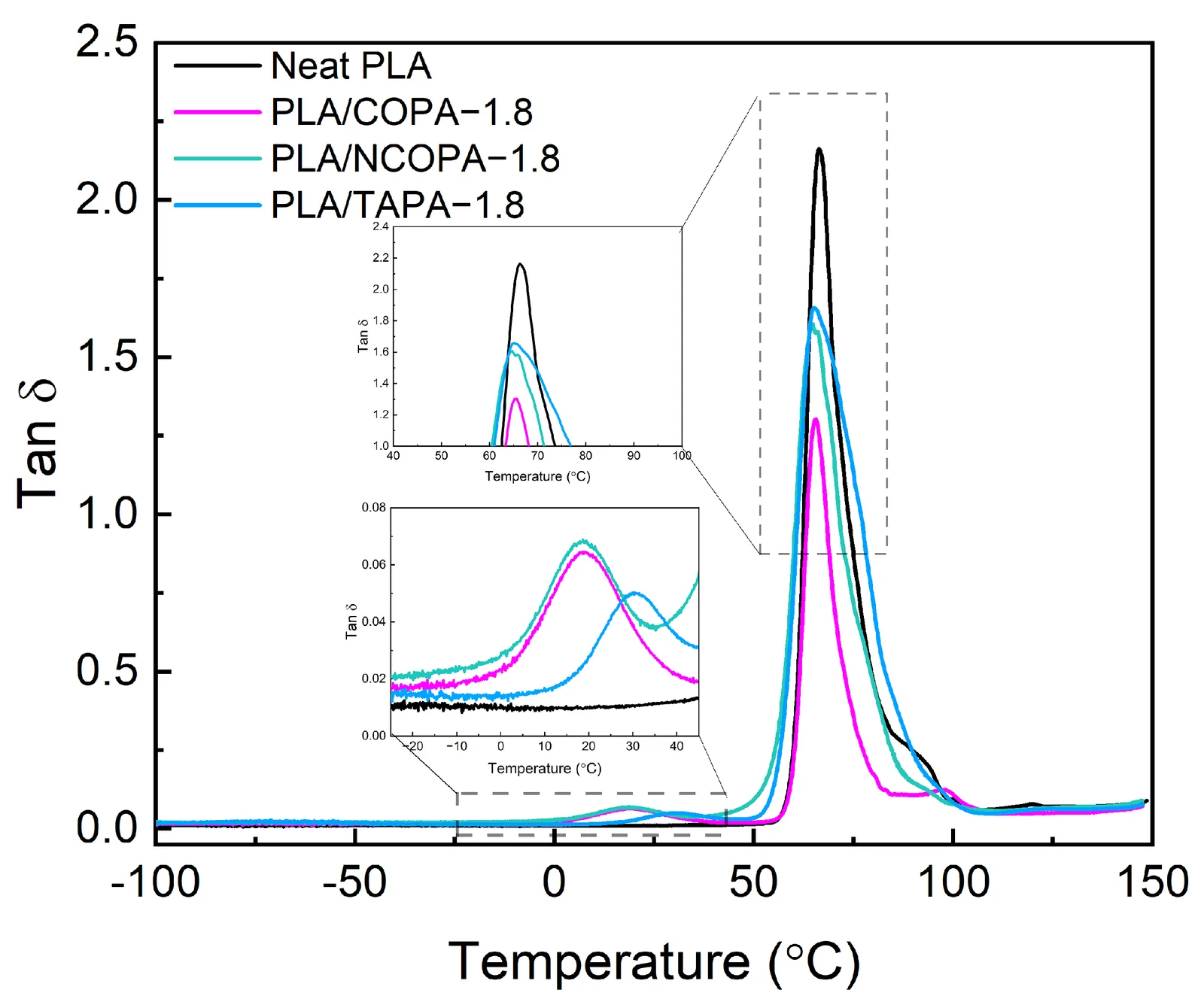
The loss factor (tan δ) for three PLA blends with different molecular architectures.

Figure 12 presents the temperature dependence of the loss factor (tan δ) of PLA blends with different molecular architectures, with relevant data presented in Table S1. The three PLA blends exhibited two distinct relaxation peaks, corresponding to the PLA and polyamide components, respectively. The PLA/COPA-1.8 and PLA/NCOPA-1.8 blends exhibited comparable *T*_g,polyamide_ values of 18.7 °C. In contrast, the *T*_g,polyamide_ of the PLA/TAPA-1.8 blend increased to 30.4 °C. This indicates that a highly cross-linked polyamide phase was formed via the reaction between HDI and TA.

Moreover, the characteristic PLA carbonyl peak at 1758 cm^−1^ was still observed in all three blends after thorough etching in CHCl_3_, demonstrating that reactive compatibilization occurred between the polyamide elastomer phase and PLA matrix. The A_C=O,PLA_/A_amide-I_ values for the PLA/COPA-1.8, PLA/NCOPA-1.8, and PLA/TAPA-1.8 blends were determined to be 0.71, 0.68, and 0.74, respectively (Figure 11c–e), providing additional evidence for favorable adhesion between PLA and polyamide.

#### 3.3.3. Morphological Evolution

**Figure 13:**
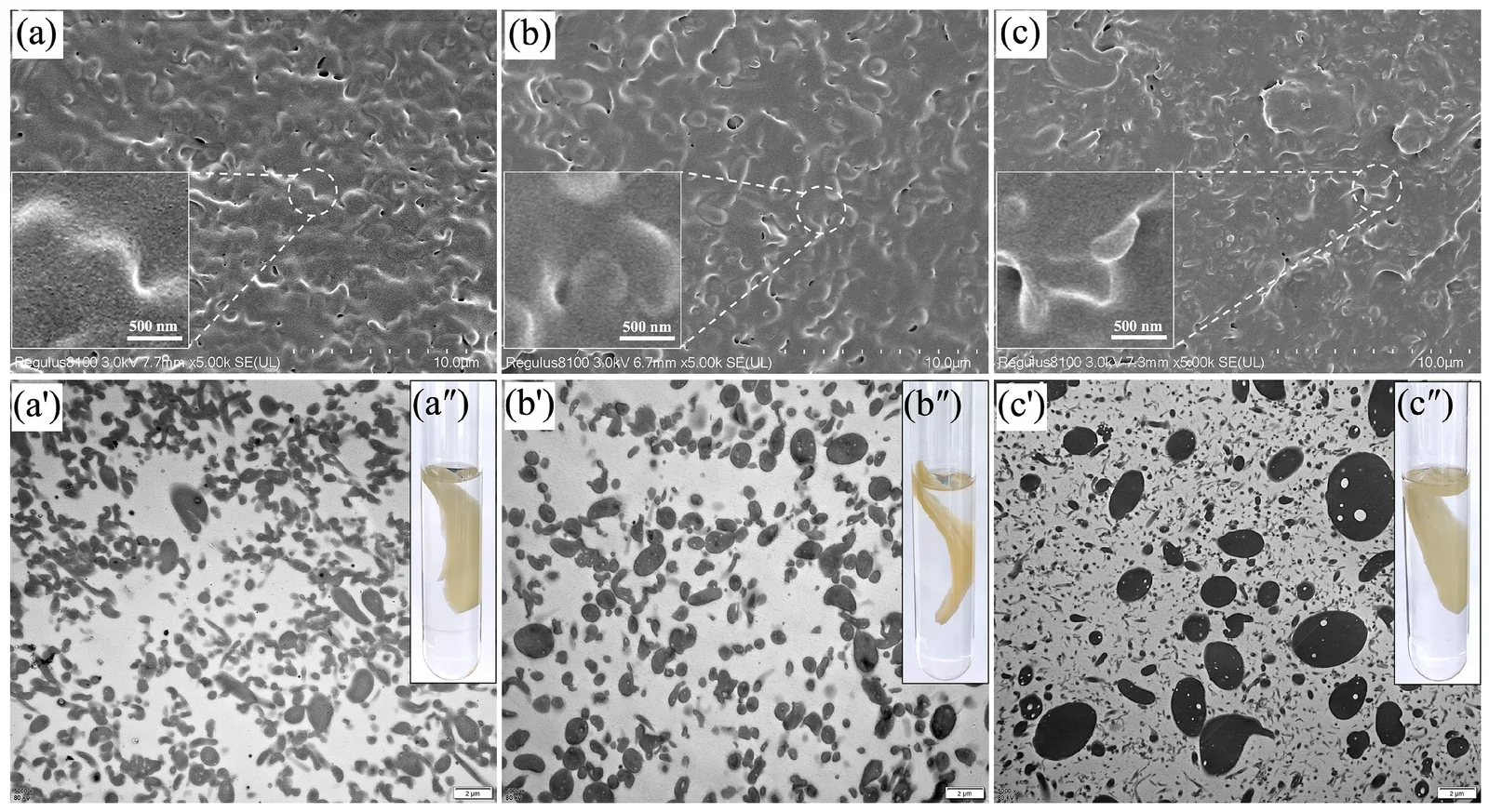
SEM images of cryo-fractured surfaces (**a**–**c**), RuO_4_-stained TEM micrographs (**a′**–**c′**), and photograph of a 1 mm thick plate specimen after 1.5 h of extraction in CHCl_3_ (**a″**–**c″**) of PLA blends with different molecular architectures: (**a**,**a′**,**a″**) PLA/COPA-1.8, (**b**,**b′**,**b″**) PLA/NCOPA-1.8, (**c**,**c′**,**c″**) PLA/TAPA-1.8.

The microstructure of the three PLA blends was investigated using SEM and TEM. As shown in Figure 13a–c, the cryo-fractured surfaces of all three blends exhibited numerous wrinkles and dispersed particles that were well-integrated into the matrix, indicating good compatibility between the PLA matrix and polyamide elastomer. Figure 13a′–c′ show the RuO_4_-stained TEM micrographs of three PLA blends. All three blends display pronounced percolation of polyamide particles into a pseudo-network morphology. To further verify the phase continuity of the three PLA blends, sheet samples were immersed in CHCl_3_ to selectively remove the PLA components (Figure 13a″–c″). Notably, after 1.5 h of immersion, all three blends almost completely retained their original shapes, indicating that the polyamide phase formed a continuous network throughout the blends.

**Figure 14:**
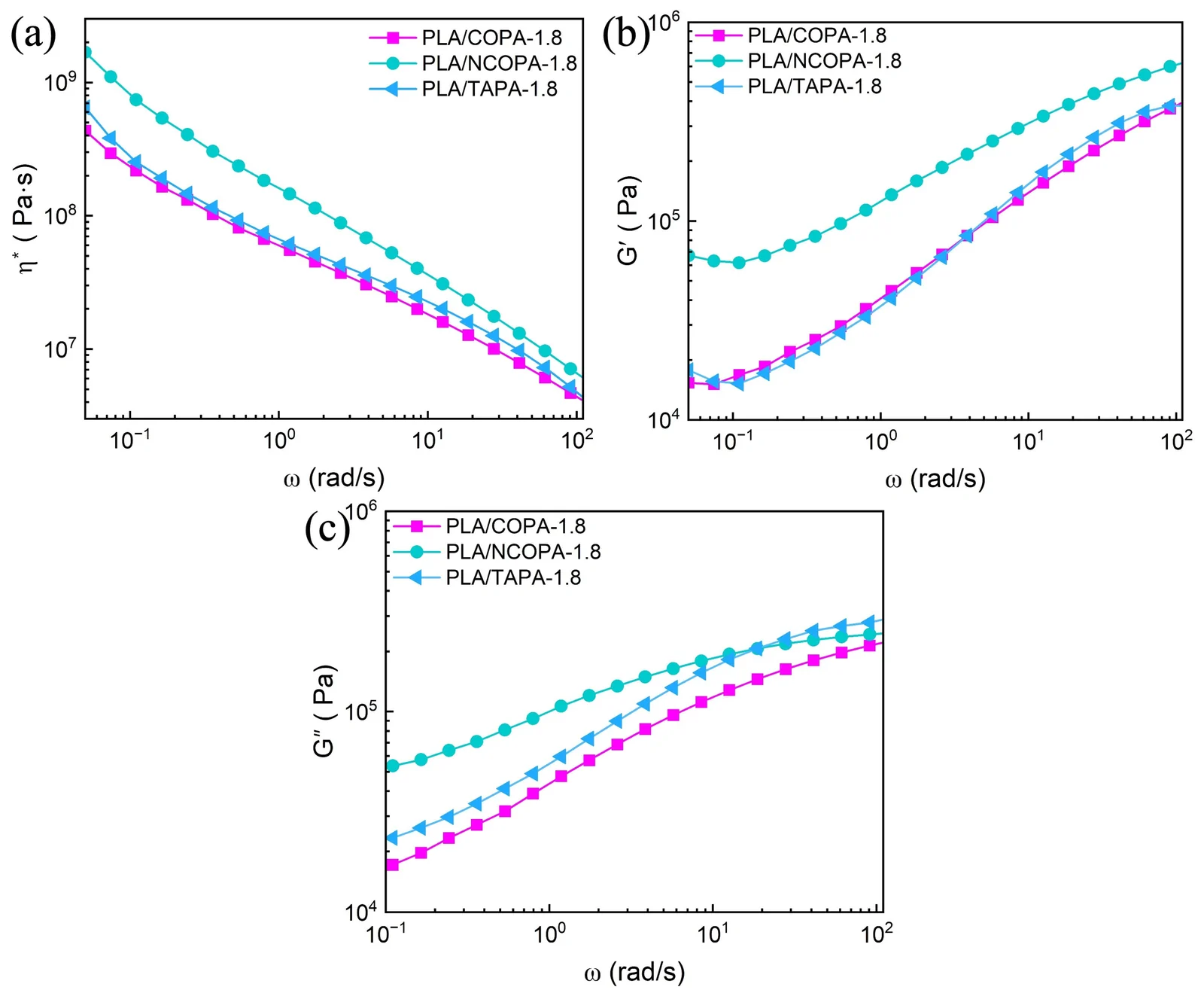
Dynamic rheological properties versus angular frequency at 185 °C for PLA blends with different molecular architectures: (**a**) complex viscosity (η*); (**b**) storage modulus (G′); (**c**) loss modulus (G″).

Rheological measurements provide a sensitive method to study the phase structure of polymer blends. Figure 14 illustrates the evolution of the complex viscosity (η*), storage modulus (G′), and loss modulus (G″) versus angular frequency for PLA blends with three different molecular architectures. As shown in Figure 14a, the three PLA blends exhibited pronounced shear-thinning behavior over the entire frequency range, which was particularly pronounced for the PLA/NCOPA-1.8 blend. The η* value of the PLA/TAPA-1.8 blend was marginally higher than that of the PLA/COPA-1.8 blend. Notably, the PLA/NCOPA-1.8 blend exhibited the highest η* values across the entire frequency range, which was attributed to the higher polyamide crosslink density and a more complete co-continuous morphology. Likewise, the G′ value of the PLA/NCOPA-1.8 blend was significantly higher than that of the other two blends. Furthermore, a low-frequency plateau was observed from the G′ versus frequency curves of all three PLA blends (Figure 14b), indicating the continuity of the highly cross-linked polyamide networks in all three blends. The Han plots and Cole–Cole plots for PLA/COPA-1.8, PLA/NCOPA-1.8, and PLA/TAPA-1.8 were shown in Figure S8. The terminal regions of the Han plots for both the PLA/COPA-1.8 and PLA/TAPA-1.8 blends lay above the isomodal line (G′ = G″) (Figure S8a). However, over the broader frequency range, the Han curve of the PLA/COPA-1.8 blend remained consistently closer to the isomodal line. Notably, the Han curve of the PLA/NCOPA-1.8 blend lay almost entirely above the isomodal line (G′ > G″) across almost the entire measured frequency range, indicating that its viscoelastic response was dominated by elasticity. A linear shape in the Cole–Cole plots was observed for all three blends, which is characteristic of their co-continuous or network-like structure (Figure S8b).

**Scheme 1:**
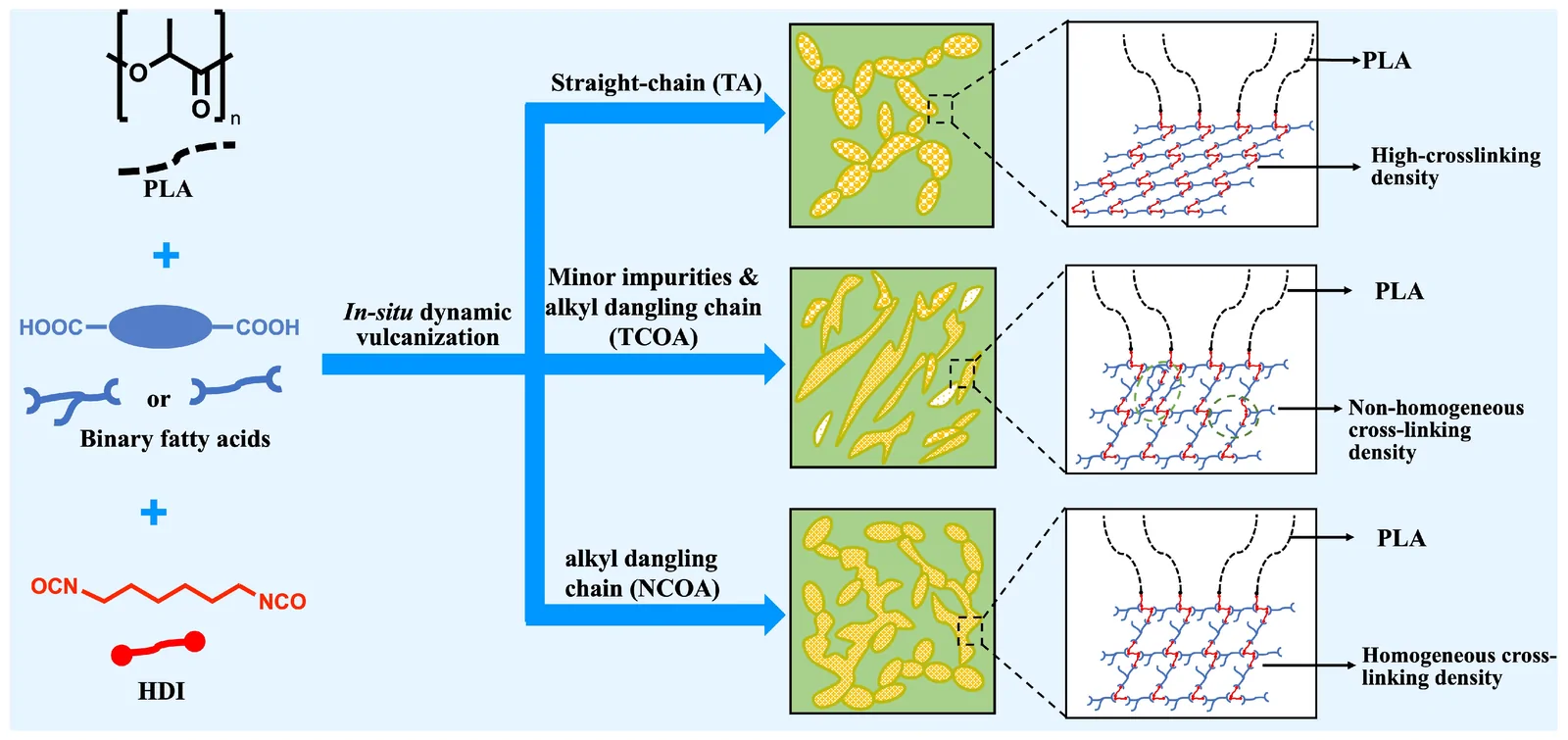
Schematic illustration of the possible mechanisms for morphological evolution in PLA blends with different molecular architectures.

**Figure 15:**
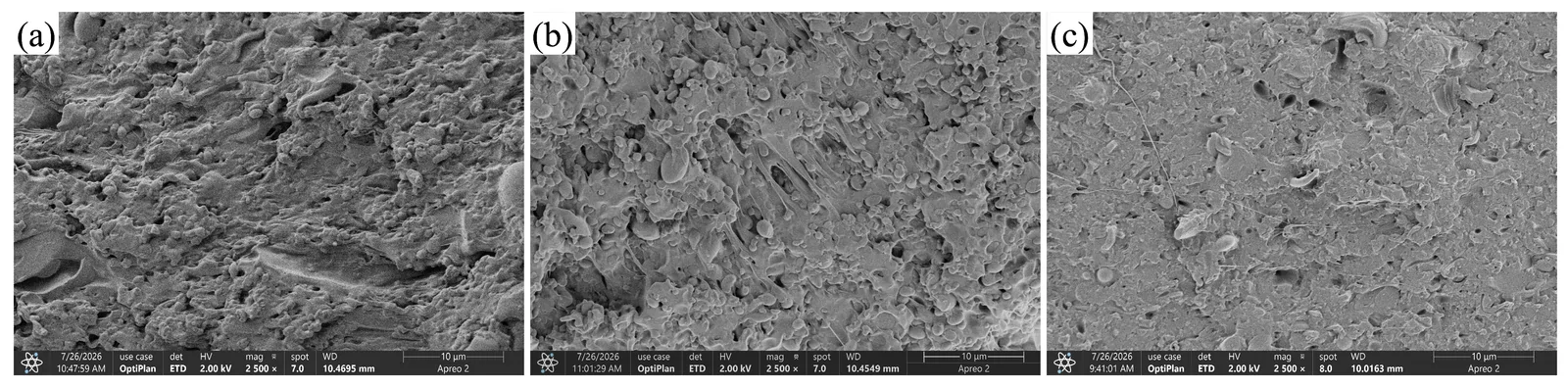
SEM micrographs of impact-fractured surfaces of PLA/COPA-1.8 (**a**), PLA/NCOPA-1.8 (**b**) and PLA/TAPA-1.8 (**c**).

Scheme 1 presents the proposed mechanisms for the morphological evolution of PLA blends with different molecular architectures. The SEM images, TEM images, and A_C=O,PLA_/A_amide-I_ area ratios confirmed that all three blends exhibited good interfacial compatibility. However, when using TA with a linear chain, the resulting TAPA elastomer exhibited an excessively high crosslink density, as evidenced by both crosslink density measurements and DMA results. This was also confirmed by the torque versus time curve. During HDI-induced dynamic vulcanization of dibasic fatty acids, the PLA/TAPA-1.8 blend exhibited a higher final torque than both the PLA/COPA-1.8 and PLA/NCOPA-1.8 blends (Figure S9 and Table S3). However, an excessively high crosslink density was detrimental to the internal cavitation of the polyamide domains, as evidenced by the SEM images of the impact-fractured surfaces (Figure 15). The fracture surface of the PLA/TAPA-1.8 blend was notably smooth and flat, with virtually no signs of plastic deformation, exhibiting only a few isolated microvoids (Figure 15c).

In contrast, when employing TCOA or NCOA with long alkyl dangling chains, the resulting polyamide elastomer possessed the optimal crosslink density, and the long alkyl dangling chains induced localized plasticization that enhanced the inter-segmental mobility of PLA. Consequently, the fracture surface of PLA/COPA-1.8 and PLA/NCOPA-1.8 blends were highly rough, with extensive plastic deformation and pronounced shear yielding. When using TCOA (synthesized from technical-grade TOA feedstock), minor impurities (mono- or trifunctional fatty acids) introduced defects or excessive crosslinking into the COPA elastomer network during dynamic vulcanization. This produced a less homogeneous network that compromised the integrity of the bicontinuous morphology in the blend. By contrast, the use of NCOA as the vulcanizing monomer enabled the formation of a more uniform and complete bicontinuous structure, which is consistent with the SEM observations of the impact-fractured surfaces. Compared with PLA/COPA-1.8, the fracture surface of PLA/NCOPA-1.8 exhibited a more distinct and complete sequence of toughening features, including a large number of cavitation voids dispersed across the entire surface, more pronounced shear yielding, and the appearance of fibrous structures (Figure 15b).

## 4. Conclusions

Two carboxylated fatty acids with different molecular structures (TCOA and NCOA) were synthesized via a thiol-ene click reaction using technical-grade and high-purity oleic acid as the starting materials. TCOA was dynamically vulcanized with hexamethylene diisocyanate (HDI) to toughen PLA. As the n_NCO,HDI_/n_COOH,TCOA_ ratio increased, the PLA/COPA blends exhibited enhanced gel content and interfacial adhesion with PLA, along with a transition in their phase morphology from a sea-island morphology to a partially or fully co-continuous architecture, revealing that the NCO/COOH ratio governs toughening performance by controlling the crosslinked network topology and the resulting phase morphology. Consequently, compared to neat PLA, the PLA/COPA-1.8 blend achieved a tensile strength of 37.0 MPa and a notched impact strength of 86.5 kJ/m^2^, representing a 20.1-fold enhancement in impact toughness. In addition, PLA blends with comparable gel contents, interfacial compatibilization, and continuous morphologies were obtained by melt-blending PLA with HDI and either TA or NCOA. Straight-chain TA reacted with HDI to in situ form polyamide elastomers with excessively high crosslink densities, thereby inhibiting internal cavitation within TAPA and leading to brittle fracture. Comparatively, using TCOA and NCOA with long alkyl dangling units yielded elastomers with an optimal network density that enabled extensive cavitation and shear yielding, the primary energy-dissipation mechanisms. However, utilizing TCOA as a toughening monomer produced defects or excessive crosslinking in the COPA elastomer network due to trace impurities (mono- or trifunctional fatty acids). This compromised the integrity of the co-continuous morphology in the blends. Therefore, the tensile modulus (2.7 GPa) and tensile strength (48.5 MPa) of the PLA/TAPA-1.8 blend were both superior to those of the PLA/COPA-1.8 and PLA/NCOPA-1.8 blends. However, its notched impact strength was the lowest, with a value of only 7.0 kJ/m^2^. The PLA/NCOPA-1.8 blend using NCOA as the toughening monomer exhibited the highest impact toughness, with a maximum notched impact strength of 131.0 kJ/m^2^. These findings highlight the crucial roles of crosslink density and network regularity of the toughening phase in the mechanical properties of PLA -based materials.

## Data Availability

The data that support the findings of this study are available from the corresponding authors upon reasonable request.

## Supplementary Materials

The following supporting information can be downloaded at: https://www.mdpi.com/article/10.3390/polym18182297/s1, Figure S1: FTIR spectra of TOA, NOA, MPA, NCOA and TCOA; Figure S2: TGA curves of NCOA and TCOA; Figure S3: Torque versus mixing time profiles of PLA blends prepared at different n_NCO,HDI_/n_COOH,TCOA_ ratios; Figure S4: Solid-state ^13^C NMR spectrum of the chloroform-insoluble residue from the PLA/COPA-1.8 blend; Figure S5: Particle size distribution of the dispersed COPA phase in the PLA/COPA-1.2 blend; Figure S6: Dynamic rheological properties versus angular frequency at 185 °C for neat PLA and PLA/COPA blends with different n_NCO,HDI_/n_COOH,TCOA_ ratios: (a) Han plots; (b) Cole-Cole plots; Figure S7: The complex viscosity (ƞ*) of the PLA/COPA-1.8 blend as functions of sweep time (30 min) at a fixed frequency of 1 rad/s and strain amplitude of 1% and temperature of 185 °C; Figure S8: Dynamic rheological properties versus angular frequency at 185 °C for PLA blends with different molecular architectures: (a) Han plots; (b) Cole-Cole plots; Figure S9: Torque versus mixing time profiles of three PLA blends with different molecular architectures; Figure S10: DSC curves of the neat PLA and PLA blends during the first heating scan; Table S1: The gel content, apparent crosslinking density and glass transition temperatures (*T*_g_) of each component of the PLA blends, and FTIR absorption peak area ratio (A_C=O,PLA_/A_amide-I_) obtained after CHCl_3_ extraction of PLA blend samples; Table S2: Mechanical data of neat PLA and PLA blends; Table S3: Reaction times, final torque values, and thermal properties of neat PLA and PLA blends; Table S4: Summary of mechanical properties for the PLA/COPA blends (this work) and representative super-toughened PLA blends from the literature.
