# Supplementary material for: Toughening PLA via Diisocyanate-Induced Dynamic Vulcanization of Carboxylated Oleic Acid: Role of Crosslinked Network Topology
Source: Polymers (Basel). 2026 Sep 19;18(18):2297. doi: 10.3390/polym18182297 (PMC13611218; doi:10.3390/polym18182297)
Supplement: Supplementary file 1 [file polymers-18-02297-s001.zip › polymers-4564564-supplementary.pdf]

Supplementary data

Toughening PLA via Diisocyanate-Induced Dynamic Vulcanization of Carboxylated Oleic Acid: Role of Crosslinked Network Topology

Dongmei Xie, Xiao Li, Jiaqi Cai, Xiaodi Mao, Hongzhi Liu*, Ping Zhang*

School of Materials Science and Engineering, NingboTech University, Ningbo 315100, China

*Corresponding authors, **E-mail**: hzliu@iccas.ac.cn (Prof. Hongzhi Liu);

zhangping@nbt.edu.cn (Prof. Ping Zhang)

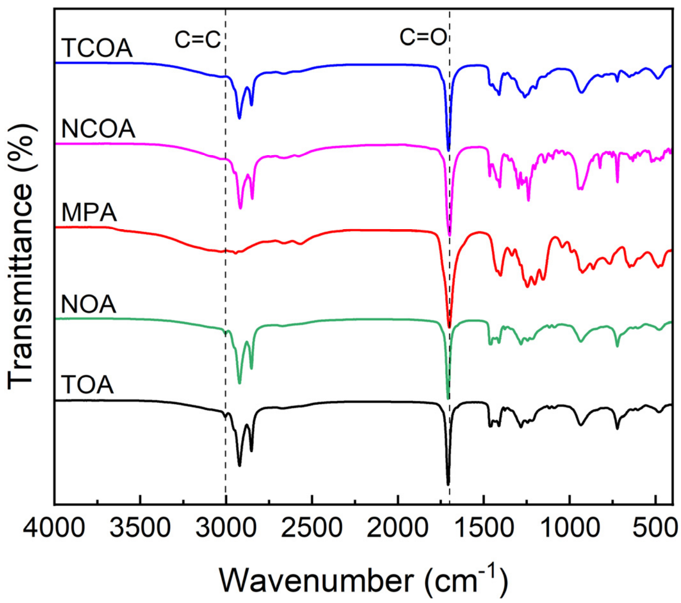

Figure S1. FTIR spectra of TOA, NOA, MPA, NCOA and TCOA.

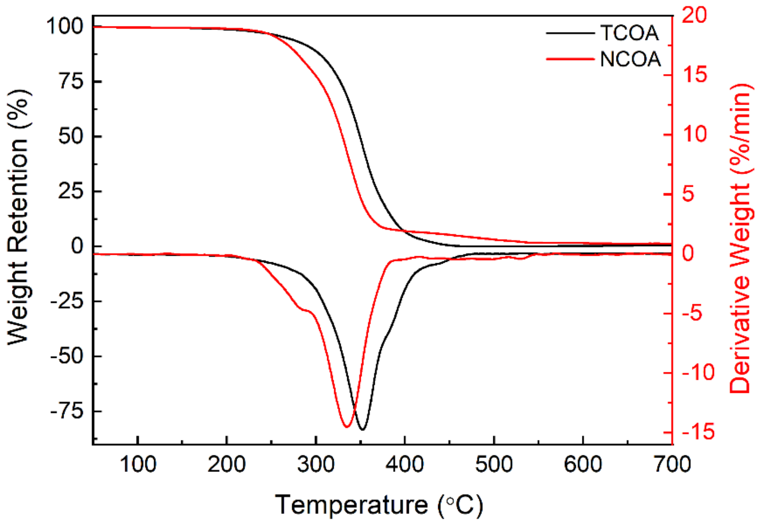

Figure S2. TGA curves of NCOA and TCOA.

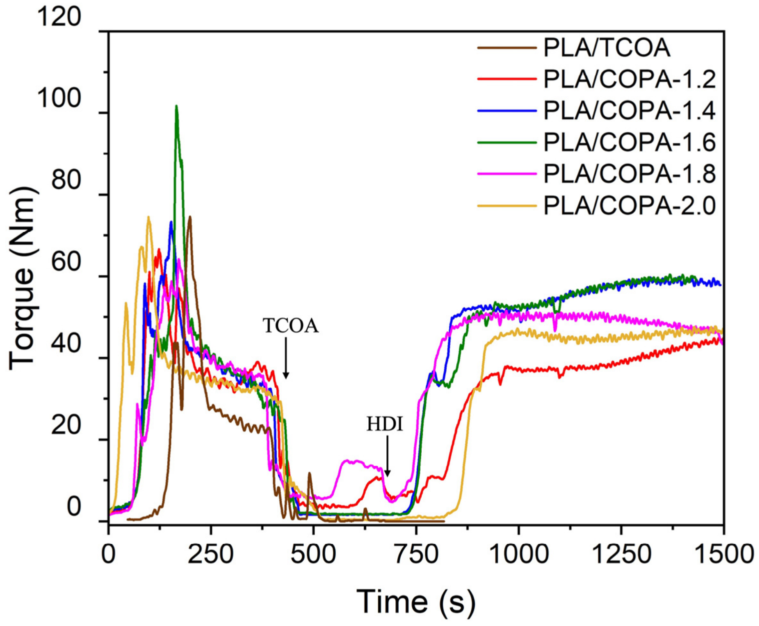

Figure S3. Torque versus mixing time profiles of PLA blends prepared at different $n_{NCO,HDI}/n_{COOH,TCOA}$ ratios.

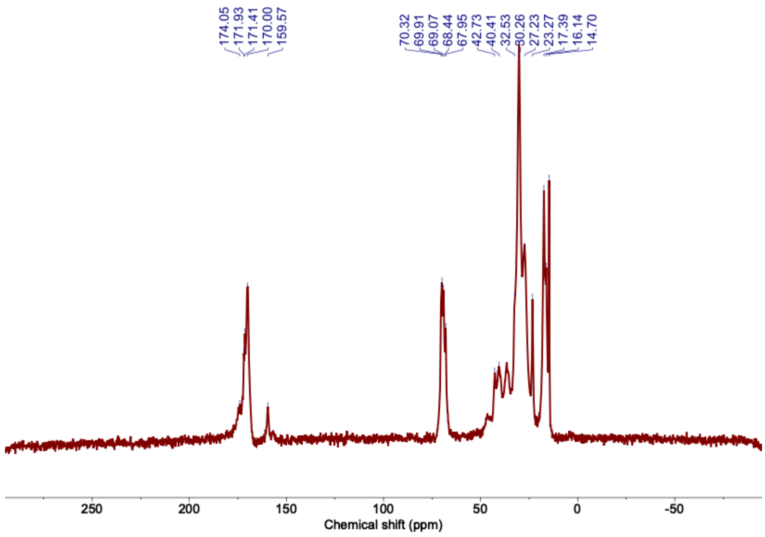

Figure S4. Solid-state ^{13}C NMR spectrum of the chloroform-insoluble residue from the PLA/COPA-1.8 blend.

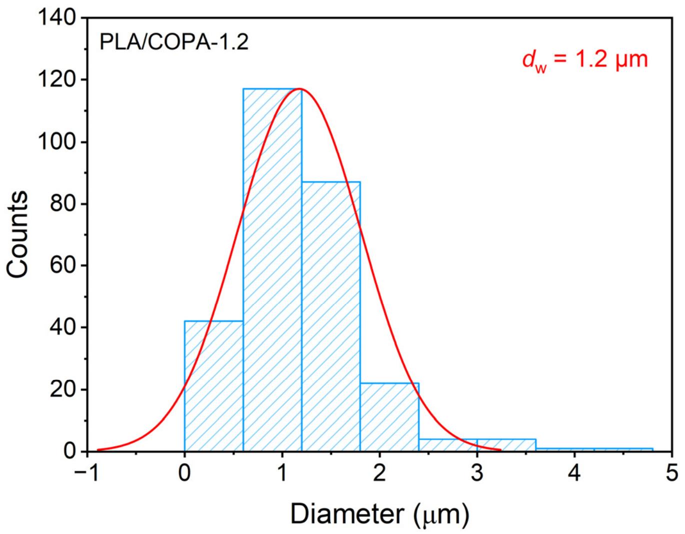

Figure S5. Particle size distribution of the dispersed COPA phase in the PLA/COPA-1.2 blend.

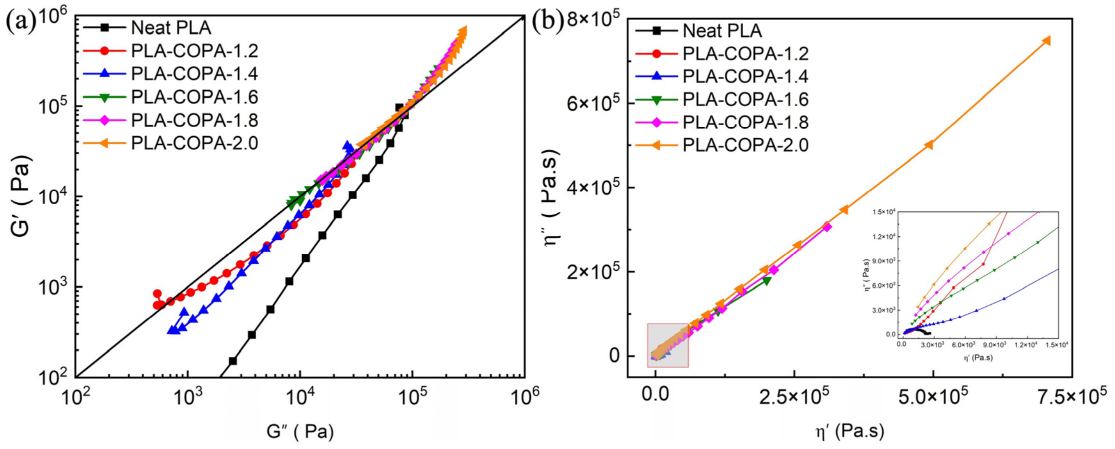

Figure S6. Dynamic rheological properties versus angular frequency at 185°C for neat PLA and PLA/COPA blends with different $n_{NCO,HDI}/n_{COOH,TCOA}$ ratios: (a) Han plots; (b) Cole-Cole plots.

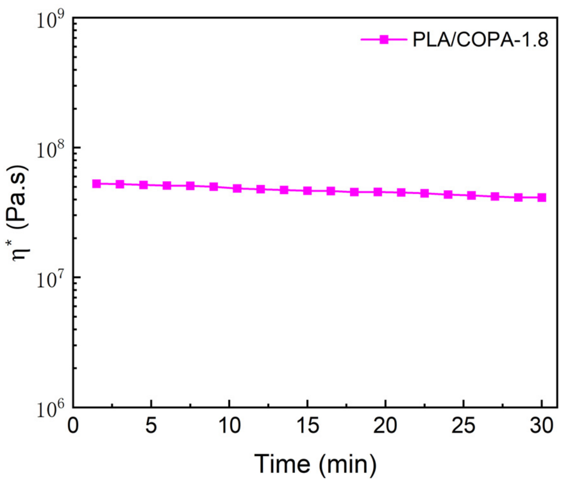

Figure S7. The complex viscosity (η*) of the PLA/COPA-1.8 blend as functions of sweep time (30 min) at a fixed frequency of 1 rad/s and strain amplitude of 1% and temperature of 185 °C.

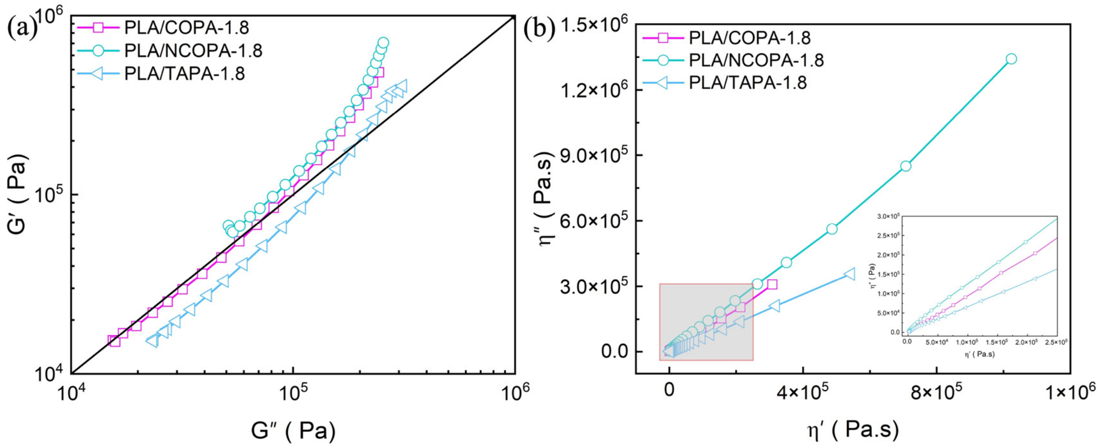

Figure S8. Dynamic rheological properties versus angular frequency at 185°C for PLA blends with different molecular architectures: (a) Han plots; (b) Cole-Cole plots.

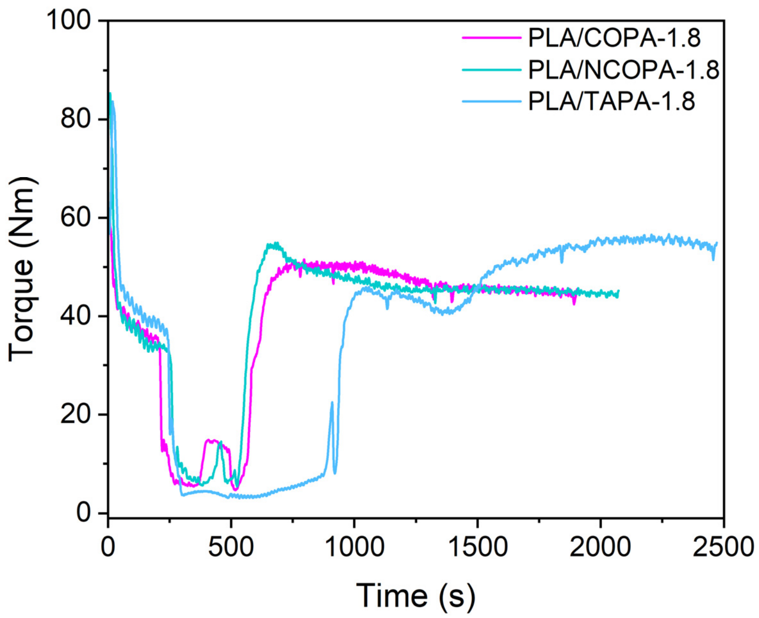

Figure S9. Torque versus mixing time profiles of three PLA blends with different molecular architectures.

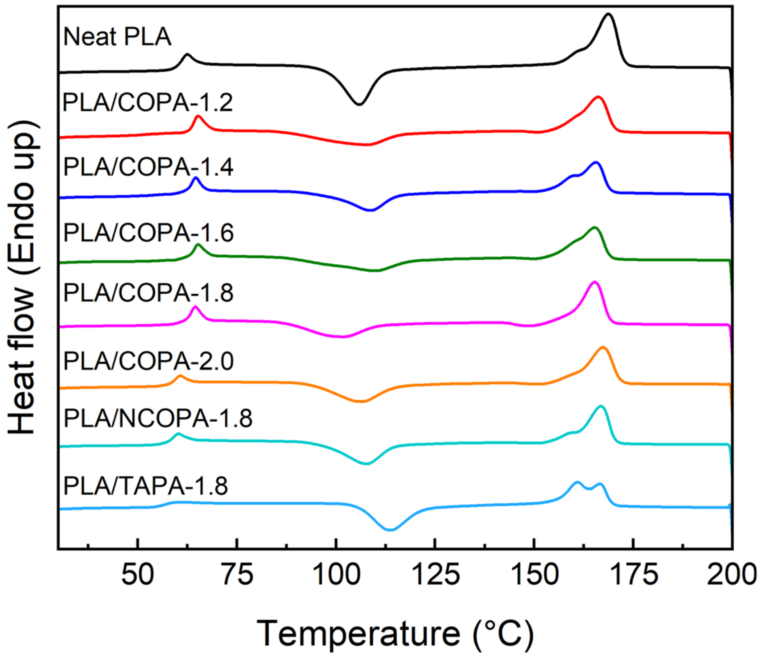

Figure S10. DSC curves of the neat PLA and PLA blends during the first heating scan.

Table S1. The gel content, apparent crosslinking density and glass transition temperatures (T_g) of each component of the PLA blends, and FTIR absorption peak area ratio ($A_{C=O,PLA}/A_{amide-I}$) obtained after $CHCl_3$ extraction of PLA blend samples.

Sample	Gel content (%)	Apparent crosslink density (V_r)	$A_{C=O,PLA}/A_{amide-I}$	$T_{g,polyamide}$ (°C)	$T_{g,PLA}$ (°C)
Neat PLA	--	--	--	--	66.3
PLA/COPA-1.2	18.9±1.1	0.39±0.002	0.22	6.7	66.3
PLA/COPA-1.4	25.7±0.8	0.46±0.009	0.31	9.5	65.8
PLA/COPA-1.6	30.6±1.6	0.51±0.007	0.64	17.5	66.6
PLA/COPA-1.8	28.5±0.5	0.52±0.001	0.71	18.7	65.5
PLA/COPA-2.0	25.0±0.3	0.56±0.047	0.67	20.6	64.1
PLA/NCOPA-1.8	27.0±0.2	0.58±0.008	0.68	18.7	64.7
PLA/TAPA-1.8	28.5±1.6	0.65±0.038	0.74	30.4	65.1

Note: $T_{g,polyamide}$ and $T_{g,PLA}$ were determined by DMA.

Table S2. Mechanical data of neat PLA and PLA blends.

Sample	Tensile Modulus (GPa)	Tensile strength (MPa)	Elongation at break (%)	Notched IS (kJ/m^2)
Neat PLA	3.6±0.5	65.7±2.1	8.5±0.5	4.3±0.3
PLA/TCOA	3.5±0.3	49.8±4.4	14.3±2.6	3.2±0.4
PLA/COPA-1.2	1.6±0.1	23.2±0.7	175.1±13.6	5.7±0.6
PLA/COPA-1.4	1.6±0.1	28.8±2.6	276.6±20.9	25.3±3.2
PLA/COPA-1.6	1.5±0.1	31.9±3.6	243.2±34.0	76.3±8.3
PLA/COPA-1.8	1.9±0.2	37.0±3.1	253.7±27.4	86.5±10.8
PLA/COPA-2.0	1.9±0.1	38.8±2.5	289.3±27.0	70.1±7.6
PLA/NCOPA-1.8	1.9±0.1	36.3±3.3	254.1±24.1	131.0±10.6
PLA/TAPA-1.8	2.7±0.3	48.5±3.6	207.7±22.0	7.0±1.1

Table S3. Reaction times, final torque values, and thermal properties of neat PLA and PLA blends.

Sample	Reaction times (min)	Final torque values (Nm)	$T_{g, PLA}$ (°C)	$T_{cc,PLA}$ (°C)	$T_{m,PLA}$ (°C)	$X_{c,PLA}$ (%)
Neat PLA	-	-	60.7	105.8	168.7	8.9
PLA/TCOA	4.5	0.2	-	-	-	-
PLA/COPA-1.2	19.3	46	63.2	107.8	166.2	8.0
PLA/COPA-1.4	16.6	59	62.6	108.8	165.7	4.7
PLA/COPA-1.6	15.0	60	63.0	110.1	165.4	3.9
PLA/COPA-1.8	27.5	45	62.3	101.6	165.3	11.0
PLA/COPA-2.0	24.4	46	58.2	111.3	167.0	5.8
PLA/NCOPA-1.8	27.7	45	57.6	107.8	166.8	4.3
PLA/TAPA-1.8	25.2	55	60.7	113.8	160.5/167.4	1.5

Table S4. Summary of mechanical properties for the PLA/COPA blends (this work) and representative super-toughened PLA blends from the literature.

systems	PLA content (%)	TS (MPa)	Notched IS （J/m）	Reference
This work	70	37.0	865	-
Neat PLA	100	65.7	43	
PLA/HDA/diisocyanate	80	36.3	801	[42]
Neat PLA	100	62.8	19	
PLA/HDAPA (LDI + HDA)	80	34.0	947.1	[40]
Neat PLA	100	56.1	35.7	
PLA/HDAPA20 (LDI + HDA)	80	34.4	1098	[41]
Neat PLA	100	56.1	36	
PLA/HPA (HDI + HDA)	75	33.3	1132	[51]
Neat PLA	100	62.0	30	
PLA /PEG/glycerol/MDI	80	43.2	408	[34]
Neat PLA	100	64.9	17	
PLA/ VESO	80	34.4	542.3	[36]
Neat PLA	100	58.4	34.1	
PLA/PMDI/PEG	70	30	546	[33]
Neat PLA	100	68.1	16	
PLA/polyurethane elastomer prepolymer (PUEP)	70	44.9	550.2	[53]
Neat PLA	100	62.2	25.5	
PLA/PBSFG	80	30.9	761	[28]
Neat PLA	100	57.6	32	
PLA/PBSePM	80	32.2	830	[29]
Neat PLA	100	63.3	29	
PLA/PCL/MDI	-	39.5	550.1	[54]
Neat PLA	100	60.4	22.1	
PLA/EBA-GMA/EMAA-Zn (80/10/10-240°C)	80	36.2	777.2	[16]
Neat PLA	100	58.2	38.0	
PLA/PO3G/HDI	80	47.77	560	[55]
Neat PLA	100	69.60	34	
PLA/PO3G-MPU	80	42.3	590	[4]
Neat PLA	100	69.5	34.3	
PLA/PPE/HDI	80	33.1	753	[31]
Neat PLA	100	62.2	20	
PLA/Pripol/HDI	80	35.1	871	[44]
Neat PLA	100	56.1	31	
PLA/UPE/DCP	80	37.5	586.6	[52]
Neat PLA	100	68.1	16.8	

PLA/UPE/PDLA/DCP	80 (PLLA/PDLA)	-	659	[56]
Neat PLA	100	-	32.2	
PLA/NR-GMA/DCP	80	~ 35	734	[21]
Neat PLA	100	~ 73	27	

Note: For consistency, IS values originally reported in J/m are converted using 1 kJ/m² = 10 J/m.
